# Nomilin Regulates Depressive‐Like Behaviors in Mice via the Ventral Part of the Lateral Septum to Bed Nucleus of the Stria Terminalis Circuit

**DOI:** 10.1111/cns.70647

**Published:** 2025-11-24

**Authors:** Liang Chen, Boli Fu, Jiaxin Liu, Cun Wang, Weijun Huang, Danhua Yuan, Chen Qing, Yao Zhang, Hao Hong

**Affiliations:** ^1^ Pukou Hospital of Chinese Medicine Affiliated to China Pharmaceutical University Nanjing China; ^2^ College of Pharmacy China Pharmaceutical University Nanjing China

**Keywords:** bed nucleus of the stria terminalis, depression, GABA_A_ receptors, LPS, Nomilin, ventral part of the lateral septum

## Abstract

**Background:**

The limitations of current clinical antidepressants and the slow progress in developing novel treatments highlight the need for rapid‐acting, long‐lasting antidepressants with minimal side effects. Nomilin, a naturally occurring limonoid compound, exhibits diverse pharmacological properties, including anti‐inflammatory and anti‐tumor activities. However, its potential antidepressant effects remain largely unclear.

**Methods:**

In this study, we used lipopolysaccharide (LPS)‐induced and chronic restraint stress (CRS)‐induced depression mouse models to verify the antidepressant effects of nomilin. The brain regions with altered activity after nomilin administration were identified using c‐fos immunofluorescence staining. Then chemogenetics, viral tracing, fiber photometry and pharmacological strategies were conducted to further investigate the neural circuit mechanisms of nomilin's antidepressant effects.

**Results:**

This study demonstrated that nomilin significantly alleviated depressive‐like behaviors and increased the excitability of GABAergic neurons in the ventral part of the lateral septal nucleus (LSv), a region exhibiting diminished activity in depressive states. Chemogenetic activation of LSv GABAergic neurons ameliorated LPS‐induced depressive‐like behaviors, whereas their inhibition attenuated the antidepressant effects of nomilin. Nomilin exerted its antidepressant effects via LSv to bed nucleus of the stria terminalis (BNST) GABAergic projections, with downstream GABA_A_ receptors playing a crucial role in regulating the LSv^GABA^ → BNST neural circuit.

**Conclusion:**

Collectively, these findings identify nomilin as a potential candidate for depression and provide novel insights into the development of antidepressant drugs.

## Graphical abstract

Nomilin ameliorates LPS‐induced depressive‐like behaviors in mice by activating LSv GABAergic neurons and the LSv^GABA^ → BNST neural circuit, with downstream modulation mediated by GABA_A_ receptors.

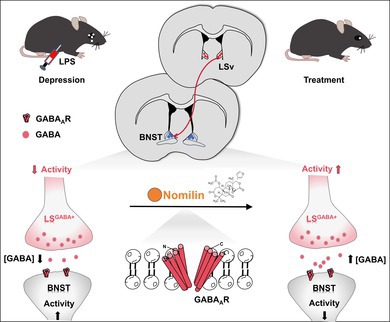

## 1 Introduction

Depression remains a major contributor to the global burden of disease, with its prevalence steadily increasing due to rising societal pressures and the frequent occurrence of stressful events. Surveys indicate that the global prevalence of depression has risen by nearly 50% over the past three decades, posing serious challenges to public health and societal stability [1, 2]. Current first‐line antidepressants, such as selective serotonin reuptake inhibitors (SSRIs), are limited by slow efficacy, low response rates, and significant side effects, often resulting in reduced patient compliance [3, 4]. Rapid‐acting antidepressants, including ketamine and zuranolone, have shown promise but are restricted by risks of addiction and limited applicability to specific populations [5, 6]. These challenges emphasize the ongoing need for the development of safe, fast‐acting, and effective antidepressant therapies.

Nomilin, a triterpenoid secondary metabolite with the molecular formula C_28_H_34_O_9_, is a natural limonoid compound extracted from commonly consumed edible citrus fruits such as lemons, citrus, and grapefruits [7]. Extensive research has demonstrated its diverse pharmacological properties, including antitumor [8, 9, 10], anti‐inflammatory [11, 12], antioxidant [13, 14], and sedative‐hypnotic activities [15]. As a natural agonist of the bile acid membrane receptor (TGR5), nomilin uniquely regulates physiological processes such as lipid and glucose metabolism [16, 17]. Notably, oleanolic acid, another natural TGR5 agonist, has been shown to improve depressive‐like behaviors in murine models [18, 19]. Previous studies have demonstrated that TGR5 expression is reduced in the lateral hypothalamic area (LHA) GABAergic neurons and dorsal hippocampal CA3 (dCA3) pyramidal neurons in mice subjected to chronic restraint stress (CRS) and chronic social defeat stress (CSDS), while its activation in LHA GABAergic neurons and the LHA^GABA^ **→** dCA3^CaMKIIα^ **→** dorsolateral septum (DLS)^GABA^ neural circuit alleviates depressive‐like behaviors [20, 21]. Despite these findings, the role of nomilin in modulating depressive states remains unexplored.

This study investigated the antidepressant effects of nomilin in lipopolysaccharide (LPS)‐induced and CRS‐induced depressive mouse models through a comprehensive approach integrating behavioral assays, viral tracing, chemogenetics, calcium signal fiber photometry, and immunofluorescence analysis. Our results identified the critical role of nomilin in modulating depressive‐like behaviors in mice and clarified the underlying neural circuit mechanisms. Overall, this study offers valuable insights into the pharmacological potential of nomilin, laying the groundwork for the development of innovative antidepressant therapies.

## 2 Materials and Methods

### 2.1 Animals

Male C57BL/6J mice (6–8 weeks, 20–25 g) were purchased from Beijing Vital River Laboratory Animal Technology Co. Ltd. (China) and male ICR mice (6–8 weeks, 35–40 g) were obtained from Jiangsu Qinglongshan Biotechnology Co. Ltd. (China). The mice were housed under controlled conditions at a temperature of 22°C ± 2°C and a humidity of 55% ± 5%, with a 12 h/12 h light–dark cycle. Both food and water were freely available. All experiments were performed in accordance with the US National Institutes of Health Guidelines and were approved by the Animal Ethics Committee of China Pharmaceutical University.

### 2.2 LPS‐Induced Depression Model

The LPS‐induced depression model was established based on previously described methods [22, 23, 24, 25]. Mice were intraperitoneally injected with LPS (1 mg/kg, Solarbio Life Science, China) for three consecutive days to induce depressive‐like behaviors.

### 2.3 CRS‐Induced Depression Model

The CRS‐induced depression model was generated following previously established protocols [20, 21, 26]. Mice were individually housed and restrained in ventilated 50 mL plastic tubes for 6 h daily (9.00 am–3.00 pm) for 21 consecutive days. The tubes were cleaned and disinfected with 75% ethanol after each use.

### 2.4 Behavioral Assessments

Mice were acclimated to the testing room for 30 min before the experiments. All tests were conducted in a quiet environment, and the apparatus was cleaned with 75% ethanol before and after use. Behavioral data were recorded and analyzed using the Animal Behavior Video Analysis Acquisition System (Jiliang, China). Heatmaps were generated using ANY‐maze software (Stoelting, USA).

#### 2.4.1 Open Field Test (OFT)

The OFT is a widely used method for assessing spontaneous activity and anxiety‐like behaviors in mice [27, 28, 29]. For the test, each mouse was placed at the center of an open field box (50 × 50 × 40 cm) and allowed to explore freely for 5 min. Video recordings were used to measure the total distance traveled and the distance covered within the central zone of the field during the last 4 min.

#### 2.4.2 Elevated Plus Maze (EPM) Test

The EPM is a classic paradigm for assessing anxiety levels in mice [30, 31, 32]. The apparatus consists of two open arms (30 × 5 cm) and two closed arms (30 × 5 × 15 cm), elevated 40–55 cm above the ground. Each mouse was placed at the intersection of the arms and allowed to explore for 5 min. Video recordings captured the time spent in open arms and the number of entries into the open arms.

#### 2.4.3 Tail Suspension Test (TST)

The TST is a widely recognized approach for evaluating behavioral despair, a hallmark of depressive‐like states in animals [33, 34, 35]. The tail of each mouse was secured with tape and suspended from a hook, ensuring the mouse remained head down at a height of 20–25 cm above the ground. Behavior was video‐recorded for 6 min, and immobility time during the final 4 min was analyzed. The different batches of mice were used for mapping the antidepressant time course of a single administration.

#### 2.4.4 Forced Swim Test (FST)

The FST is a standard procedure for assessing depressive‐like behaviors in animals [35, 36, 37]. Here, each mouse was individually placed in a transparent Plexiglass cylinder (12 cm diameter, 33 cm height) filled with water maintained at 22°C–25°C, ensuring a depth of 20–25 cm to prevent contact with the bottom. Mice were observed for 6 min, and immobility time during the last 4 min was video‐recorded and analyzed. The water was replaced after each test, ensuring consistent temperature and depth.

#### 2.4.5 Sucrose Preference Test (SPT)

The SPT is commonly employed to evaluate anhedonia, a hallmark symptom of depression [38, 39, 40, 41, 42]. Each mouse was individually housed and habituated to two bottles of water for 24 h, followed by two bottles of 1% sucrose solution for an additional 24 h. After a 24‐h period of water deprivation, the mice were given access to one bottle of water and one bottle of 1% sucrose for 2 h. To prevent positional bias, the positions of the bottles were alternated every 1 h. The sucrose preference index was determined as the percentage of sucrose intake relative to total fluid consumption.

#### 2.4.6 Conditioned Place Preference (CPP)

The CPP paradigm is a classic model for evaluating drug‐induced psychological dependence [43, 44, 45, 46]. The apparatus included a central chamber (10 × 24 × 30 cm) and two conditioning chambers (24 × 24 × 30 cm) distinguished by distinct colors and floor textures. Mice were first placed into the central chamber and allowed to explore the apparatus for 15 min to establish baseline preferences. The CPP score was calculated as the time spent in the streak‐patterned chamber minus the time spent in the gray‐patterned chamber, with a score below zero representing a preference for the gray chamber. On the first day of training, mice injected with nomilin were placed in the non‐preferred chamber for 30 min. On the second day, mice received vehicle injections and were confined to the preferred chamber for 30 min. After four training sessions, the mice were allowed to explore the entire apparatus freely for 15 min, and the CPP score was recalculated based on the time spent in each chamber.

### 2.5 Adeno‐Associated Virus (AAV) Injection

The AAVs used in this study were purchased from BrainVTA (Wuhan, China), including AAV2/9‐GAD67‐hM3Dq‐mCherry, AAV2/9‐GAD67‐hM4Di‐mCherry, AAV2/9‐GAD67‐mCherry, AAV2/9‐GAD67‐GCaMP6s‐EGFP, AAV2/9‐DIO‐hM3Dq‐mCherry, AAV2/9‐DIO‐hM4Di‐mCherry, and Retro‐AAV‐GAD67‐Cre‐EGFP.

Mice were anesthetized with isoflurane (RWD Life Science, China) and secured on a stereotaxic frame (Harvard Apparatus, USA). Following the removal of connective tissue with 3% hydrogen peroxide, the skull was exposed, taking bregma as the coordinate zero point. Small craniotomies were drilled above the target brain regions. Using a 10‐mL microsyringe (Gaoge, China) connected to a 27‐G inner syringe (RWD Life Science), the viruses (200 nL) were bilaterally injected into the ventral part of the lateral septal nucleus (LSv) (anteroposterior (AP): 0.50 mm from bregma; mediolateral (ML): ±1.00 mm; dorsoventral (DV): −3.50 mm) or the bed nucleus of the stria terminalis (BNST) (AP: 0.26 mm; ML: ±1.00 mm; DV: −4.00 mm). The injection was delivered at a rate of 100 nL/min, and the needle was left in place for 5 min to ensure adequate drug diffusion and avoid backflow. Behavioral experiments were conducted 3 weeks post‐injection to allow sufficient viral expression.

### 2.6 Cannula Implantation

Cannula implantation was performed under isoflurane anesthesia, with mice positioned on a stereotaxic frame. The cannulas (27 G, RWD Life Science) were implanted 0.1 mm above the BNST and LSv and secured with two stainless steel screws and dental cement to facilitate localized drug delivery. Mice were allowed to recover for 1 week following surgery before drug administration commenced.

### 2.7 Fiber Photometry

The fiber photometry was conducted following protocols established in previous studies ([38, 39, 47]). AAV2/9‐GAD67‐GCaMP6s‐EGFP was unilaterally injected into the LSv, and a customized optical fiber (Inper, China) was inserted above the BNST 3 weeks after the viral injection. Calcium signals were recorded using the Inper fiber photometry system (China) during the TST, following a 7‐day recovery period after optical fiber implantation. Fluorescence signals from GCaMP6s‐expressing cells were excited with a 488‐nm LED, while a 405‐nm LED served as the control. The start of mice struggling was defined as time point 0 s, and the signal values between −2 and 0 s were used to establish the baseline. Fluorescence changes (ΔF/F) were calculated using the formula (F − F0)/F0. Data were processed and analyzed using Inper Data Process and MATLAB software.

### 2.8 Drug Administration

Mice were administered ketamine (10 mg/kg, provided by Nanjing Municipal Ministry of Public Security) and clozapine‐*N*‐oxide (CNO, 2 mg/kg, Sigma, USA) via intraperitoneal injection. Nomilin (10, 20, and 40 mg/kg, Yuanye, China) was delivered by oral gavage. The GABA_A_ receptor antagonist picrotoxin (PTX, 100 μM in 200 nL, Sigma, USA) was infused locally into the BNST using a microinjection pump (Harvard Apparatus, USA) at a rate of 100 nL/min through the implanted cannulas. Similarly, CNO (3 μM in 200 nL, Sigma) was microinjected into the BNST to modulate neural circuit activity. The TGR5 antagonist SBI‐115 (1.5 μg in 200 nL, MCE, China) was microinjected into the LSv. To ensure optimal drug diffusion and prevent backflow, the injection needle was left in place within the cannula for an additional 5 min post‐infusion.

### 2.9 Immunofluorescence

Following anesthesia with isoflurane, mice were subjected to cardiac perfusion with phosphate‐buffered saline (PBS), followed by fixation with 4% paraformaldehyde. Whole brains were harvested, post‐fixed in 4% paraformaldehyde overnight, and dehydrated in 30% sucrose in PBS.

For single staining of c‐fos, frozen coronal slices (25 mm thick) were prepared using a freezing microtome (Leica, Germany). Sections were blocked with 10% donkey serum (Solarbio Life Science, China) and 0.3% Triton X‐100 (Beyotime, China) at room temperature (RT) for 2 h, followed by overnight incubation at 4°C with rabbit anti‐c‐fos primary antibodies (1:500, Synaptic Systems, Germany). After washing with PBS (3 × 8 min), the sections were incubated with Alexa Fluor 488‐anti‐rabbit secondary antibodies (1:500, Yeasen Biotechnology, China) in the dark at RT for 1 h.

For double immunofluorescent staining, paraffin‐embedded coronal slices (8 mm thick) were prepared using a paraffin microtome (KEDEE, China). After paraffin removal at 60°C for 1 h, the sections were sequentially processed through xylene, gradient ethanol, and pure water. Antigen retrieval was performed by heating sections in a microwave oven with Tris‐EDTA (Servicebio, China). After cooling to RT and washing with PBS (3 × 5 min), the sections were blocked and incubated with primary antibodies (rabbit anti‐c‐fos, 1:1000, Abcam, USA and mouse anti‐GABA, 1:400, Merck, Germany). Corresponding secondary antibodies (Alexa Fluor 488‐anti‐mouse and Alexa Fluor 594‐anti‐rabbit, 1:500, Yeasen Biotechnology) were used to conjugate with primary antibodies.

All slices were counterstained with 4′,6‐diamidino‐2‐phenylindole (DAPI, Solarbio Life Science, China) to label nuclei and imaged using a fluorescence microscope (Leica, Germany). Quantitative analyses were performed using NIH ImageJ software.

### 2.10 Statistical Analysis

All statistical analyses were performed using GraphPad Prism v9.0.0 (GraphPad Software, USA). All data underwent normality testing using the Shapiro–Wilk test or Kolmogorov–Smirnov test. Statistical analysis was performed using a two‐tailed unpaired Student's *t*‐test for comparing two groups with normally distributed data. One‐way analysis of variance (ANOVA) with Dunnett's *post hoc* test was used for comparing multiple groups containing normally distributed data or a Kruskal–Wallis test with a Dunn's multiple comparisons test for comparing multiple groups containing nonparametric distributed data. For comparison of multiple groups of more than two variables, a two‐way ANOVA followed by Bonferroni's *post hoc* multiple comparisons test or Fisher's least significant difference (LSD) test was used. Data are expressed as mean ± standard error of the mean (SEM). Boxplot data are presented as median, quartile, and extremum values. Significance levels are presented as ns *p* > 0.05, *p < 0.05, **p < 0.01, ***p < 0.001, ****p < 0.0001.

## 3 Results

### 3.1 Nomilin Ameliorates LPS‐ and CRS‐Induced Depressive‐Like Behaviors in Mice

**FIGURE 1:**
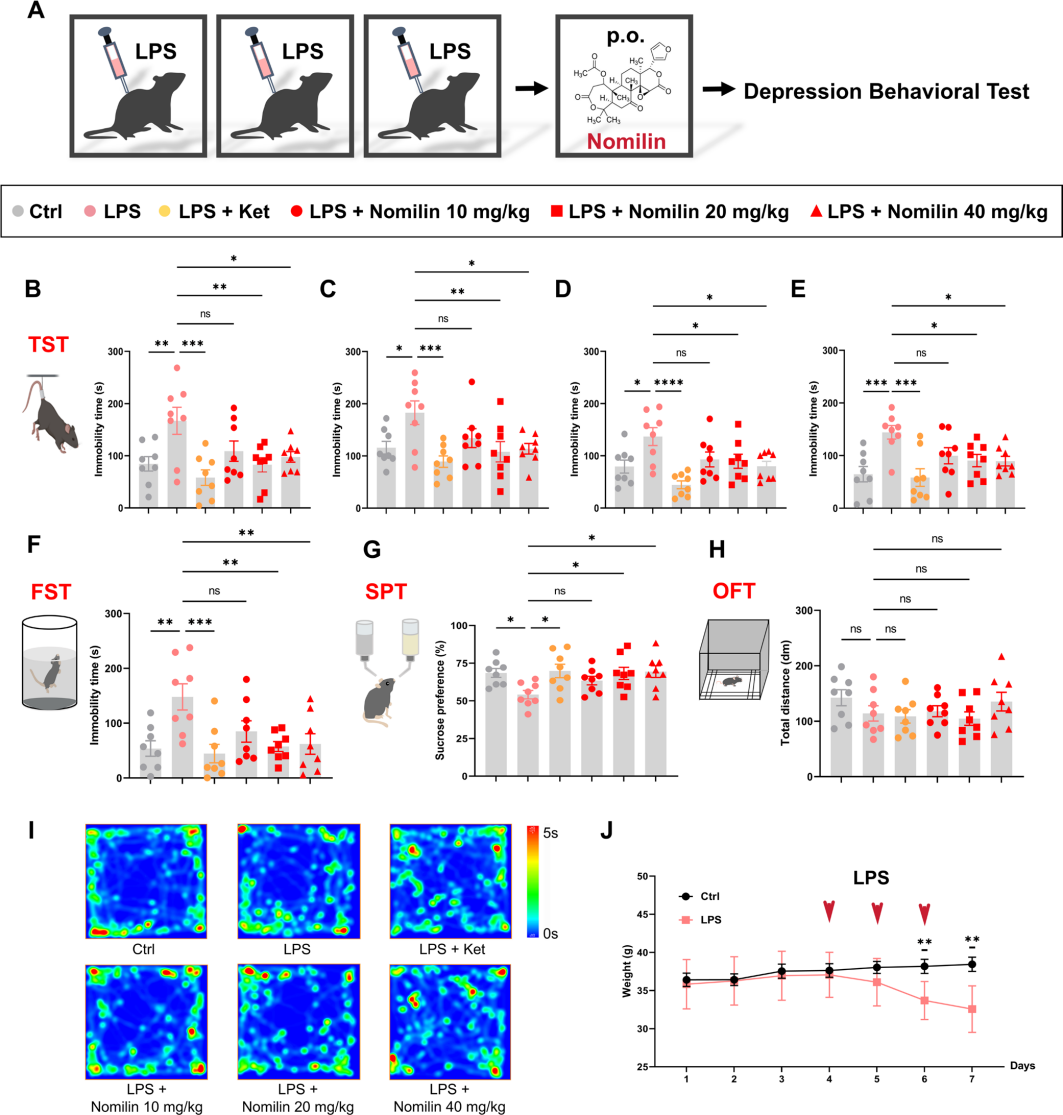
Nomilin ameliorates LPS‐induced depressive‐like behaviors in mice. (A) Experimental design. (B–E) Immobility time in the TST measured at 1 h (B), 2 h (C), 4 h (D), and 24 h (E) post‐administration (*n* = 8 mice/group from different batches; One‐way ANOVA with Dunnett's *post hoc* test; F_5, 42_ = 4.687, *p* = 0.0017 for 1 h; F_5, 42_ = 3.962, *p* = 0.0049 for 2 h; F_5, 42_ = 5.450, *p* = 0.0006 for 4 h; F_5, 42_ = 5.043, *p* = 0.0010 for 24 h). (F) Immobility time in the FST (*n* = 8 mice/group; One‐way ANOVA with Dunnett's *post hoc* test, F_5, 42_ = 4.697, *p* = 0.0017). (G) Sucrose preference in the SPT (*n* = 8 mice/group; One‐way ANOVA with Dunnett's *post hoc* test, F_5, 42_ = 2.886, *p* = 0.0250). (H, I) Total distance traveled (H) and representative heatmaps (I) in the OFT (*n* = 8 mice/group; One‐way ANOVA with Dunnett's *post hoc* test, F_5, 42_ = 1.267, *p* = 0.2960). (J) Body weight measurements of mice (*n* = 8 mice/group; Two‐tailed unpaired Student's *t‐*test; t (14) = 3.530, *p* = 0.0035 for Day 6; t (14) = 4.126, *p* = 0.0010 for Day 7). Data are presented as mean ± SEM. Significance levels: Ns *p* > 0.05; ^*^ *p* < 0.05; ^**^ *p* < 0.01; ^***^ *p* < 0.001. Ctrl, control; FST, forced swim test; Ket, ketamine; LPS, lipopolysaccharide; OFT, open field test; SPT, sucrose preference test; TST, tail suspension test.

The antidepressant effects and duration of action of nomilin were first evaluated using LPS‐ and CRS‐induced depressive‐like mouse models. Intraperitoneal injections of LPS for three consecutive days induced depressive‐like behaviors in mice, characterized by a continuous reduction in body weight from days 5 to 7 (Figure 1J). Ketamine (10 mg/kg), known for its rapid antidepressant effects [48], served as a positive control. Nomilin was administered via gavage at doses of 10, 20, and 40 mg/kg, and depressive‐like behaviors were assessed at 1, 2, 4, and 24 h post‐administration using the TST. The LPS‐treated mice exhibited significantly prolonged immobility in the TST compared to the control group. Ketamine treatment markedly reduced immobility at all time points, as did nomilin at 20 and 40 mg/kg (Figure 1A–E).

To further evaluate the antidepressant effects of nomilin, FST and SPT were performed. Results showed that both ketamine (10 mg/kg) and nomilin (20 and 40 mg/kg) significantly reduced immobility in the FST (Figure 1F) and increased sucrose preference in the SPT (Figure 1G) without affecting total fluid intake (Figure S1A) at 1 h post‐administration. Notably, no significant differences were observed in total distance traveled in the OFT between groups (Figure 1H,I), suggesting no impact on locomotor activity. These findings indicate that a single oral administration of nomilin at 20 or 40 mg/kg effectively alleviates LPS‐induced depressive‐like behaviors in mice, with the antidepressant effects of 40 mg/kg nomilin persisting for 24 h.

To assess whether nomilin exhibits universal antidepressant effects across depressive‐like states, the 21‐day CRS paradigm was employed to induce depressive‐like behaviors and weight loss in mice (Figure S2A,F). Mice treated with 10 mg/kg ketamine or 40 mg/kg nomilin for three consecutive days displayed significantly reduced immobility in the TST (Figure S2B) and FST (Figure S2C), along with increased sucrose consumption in the SPT (Figure S2D) and unchanged total water uptake (Figure S1B), compared to the control mice. Notably, spontaneous activity remained unaffected, as evidenced by unchanged OFT results (Figure S2E). These data demonstrate that 40 mg/kg nomilin also effectively ameliorates CRS‐induced depressive‐like behaviors in mice.

To evaluate the potential impact of nomilin on natural reward preference, locomotor activity, or anxiety‐like behaviors, normal mice were administered different doses of nomilin via gavage and subjected to CPP, SPT, OFT, and EPM. No significant differences in natural preference were observed, as indicated by the unchanged CPP scores and velocities (Figure S3A–E) and stable sucrose preference in the SPT Figures S3H and S1C. Similarly, nomilin treatment did not affect total or central distances traveled in the OFT (Figure S3F,G) or the time spent in or entries into the open arms in the EPM (Figure S3I,J). Taken together, these findings indicate that nomilin does not influence natural reward preference, spontaneous activity, or anxiety‐like behaviors in mice.

Our previous studies have shown that TGR5 can regulate depression‐like behaviors in mice, and the TGR5‐specific agonist INT‐777 has a significant antidepressant effect [20, 21]. Therefore, to investigate whether TGR5 is associated with nomilin's antidepressant effects, we first implanted a cannula in LSv. After 7 days of recovery, intraperitoneal injection of LPS was performed. Then TGR5 antagonist SBI‐115 (1.5 μg in 200 nL/side) was injected into LSv through the cannula for three consecutive days, and nomilin or vehicle was administered to LPS‐treated mice 1 h before behavioral tests Figure S4A–C. The results showed that nomilin treatment effectively alleviated depressive‐like behaviors in LPS‐treated mice, and intra‐LSv injection of SBI‐115 blunted the antidepressant‐like effects of nomilin, as evidenced by increased immobility in the TST and FST, as well as reduced sucrose consumption in the SPT. The total distance moved in OFT remained unchanged Figures S4D–G, S1D. We also used LC–MS/MS to detect the intracerebral concentration 1 h after gavage administration of nomilin at the dose of 20 mg/kg. The results showed that the concentration of nomilin in the brain was 23.17 ± 8.89 ng/g of brain tissue, indicating that nomilin can cross the blood–brain barrier. These results demonstrate that nomilin exerts antidepressant effects by activating TGR5 in the brain. In addition, we detected IL‐6, IL‐1*β* and TNF‐α in the brain using enzyme‐linked immunosorbent assay (ELISA). The results showed that LPS caused an increase in the expression of inflammatory factors such as IL‐6, IL‐1*β* and TNF‐*α* in the prefrontal cortex (PFC) and hippocampus (HPC), while gavage nomilin could reduce their expression but there was no statistical difference compared with the LPS group Figure S5A,B. This indicates that the antidepressant effect of nomilin has little to do with its anti‐inflammatory properties.

### 3.2 Nomilin Enhances GABAergic Neuronal Excitability in the LSv of LPS‐Induced Depressive‐Like Mice

**FIGURE 2:**
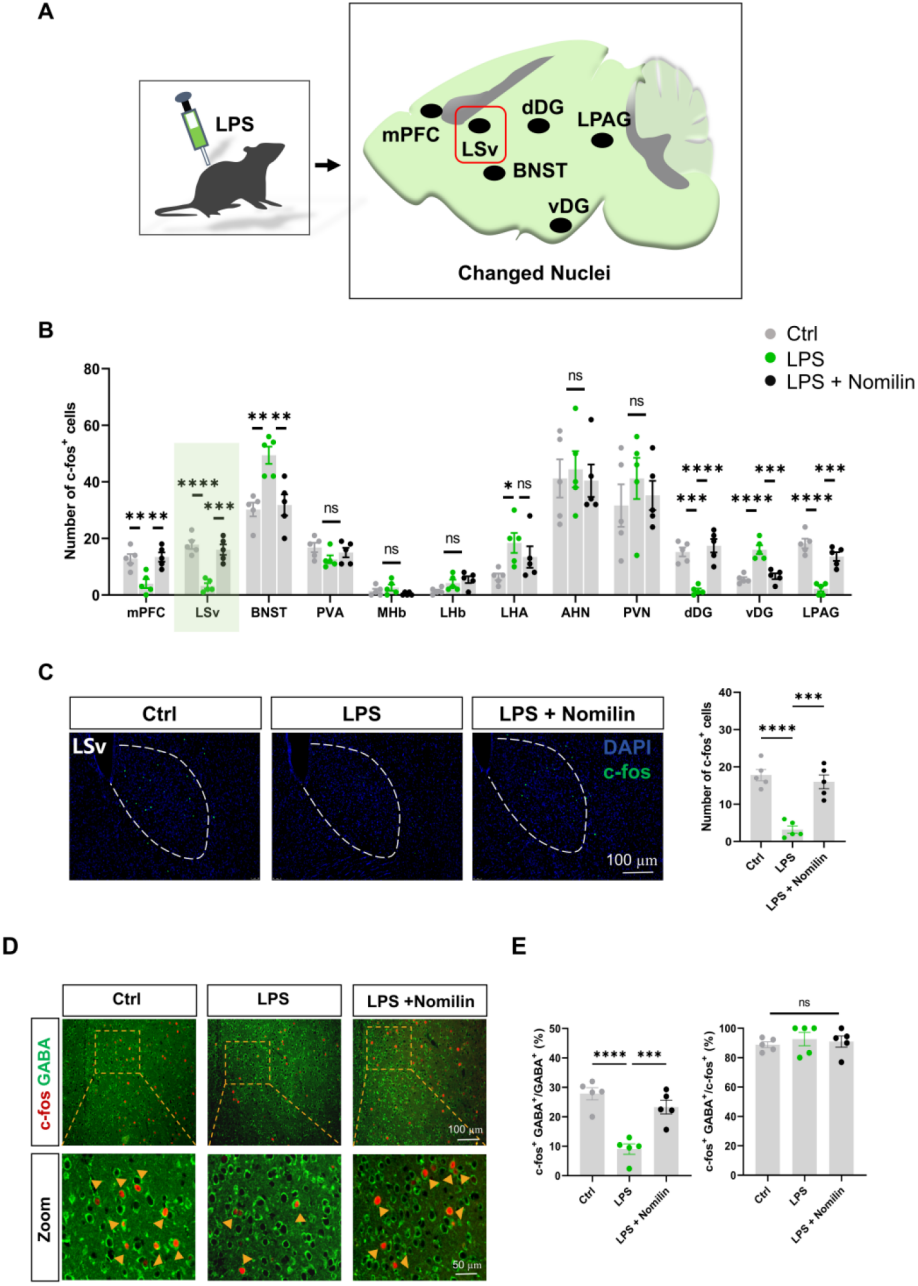
Nomilin enhances GABAergic neuronal excitability in the LSv of LPS‐treated mice. (A) Summary plot illustrating changes in c‐fos expression across nuclei in LPS‐treated mice. (B) Quantitative analysis of c‐fos^+^ cell counts in different brain regions (*n* = 5 mice/group; Kruskal–Wallis test with Dunn's multiple comparisons test for MHb; One‐way ANOVA with Dunnett's *post hoc* test for others; F_2, 12_ = 10.52, *p* = 0.0023 for mPFC; F_2, 12_ = 28.46, *p* < 0.0001 for LSv; F_2, 12_ = 11.68, *p* = 0.0015 for BNST; F_2, 12_ = 1.753, *p* = 0.2149 for PVA; *p* = 0.2124 for MHb; F_2, 12_ = 4.000, *p* = 0.0467 for LHb; F_2, 12_ = 3.841, *p* = 0.0514 for LHA; F_2, 12_ = 0.1125, *p* = 0.8945 for AHN; F_2, 12_ = 0.5179, *p* = 0.6085 for PVN; F_2, 12_ = 25.37, *p* < 0.0001 for dDG; F_2, 12_ = 25.19, *p* < 0.0001 for vDG; F_2, 12_ = 33.70, *p* < 0.0001 for LPAG). (C) Left: Representative images of c‐fos expression in the LSv. Right: Quantification of c‐fos^+^ cells in the LSv (*n* = 5 mice/group; One‐way ANOVA with Dunnett's *post hoc* test, F_2, 12_ = 28.46, *p* < 0.0001). (D) Representative images of colocalization of c‐fos^+^ and GABA^+^ cells in the LSv. (E) Quantification of percentage of colocalized c‐fos^+^ and GABA^+^ cells in total GABA^+^ or c‐fos^+^ cell populations in the LSv (*n* = 5 mice/group; One‐way ANOVA with Dunnett's *post hoc* test, F_2, 12_ = 22.32, *p* < 0.0001 for GABA^+^; Kruskal–Wallis test with Dunn's multiple comparisons test, *p* = 0.6432 for c‐fos^+^). Data are presented as mean ± SEM. Significance levels: Ns *p* > 0.05; ^*^ *p* < 0.05; ^**^ *p* < 0.01; ^***^ *p* < 0.001; ^****^ *p* < 0.0001. AHN, anterior hypothalamic nucleus; BNST, bed nucleus of the stria terminalis; dDG, dorsal dentate gyrus; LHA, lateral hypothalamic area; LHb, lateral habenula; LPAG, lateral periaqueductal gray; LSv, ventral part of the lateral septum; MHb, medial habenula; mPFC, medial prefrontal cortex; PVA, anterior paraventricular nucleus of the thalamus; PVN, paraventricular nucleus; vDG, ventral dentate gyrus.

C‐fos is a well‐established marker of neuronal activity, with its expression increasing in response to diverse stimuli in vitro and in vivo [49, 50]. Labeling activated neurons with c‐fos enables the mapping of nuclei and neural circuits involved in specific behaviors, sensory processing, learning, memory, and responses to external stimuli [51]. To explore the effect of nomilin on neuronal activity in mood‐related brain regions, c‐fos expression was analyzed in LPS‐ and CRS‐induced depressive‐like mice following nomilin administration. Immunofluorescence analysis revealed reduced c‐fos expression in the medial prefrontal cortex (mPFC), LSv, dorsal dentate gyrus (dDG), and lateral periaqueductal gray (LPAG) of LPS‐treated mice. However, treatment with nomilin significantly increased c‐fos expression in these regions (Figure 2A–C). Similarly, CRS‐induced depressive‐like mice displayed reduced c‐fos expression, which was restored by nomilin in the mPFC, LSv, dDG, and LPAG (Figure S6A–C). Among these regions, the LSv exhibited the most pronounced changes in neuronal activity and emotional relevance. LSv neurons were inhibited in both LPS‐ and CRS‐induced depressive‐like mice but showed robust activation after nomilin administration.

To identify the neuronal subtypes activated by nomilin in the LSv, genome‐wide high‐resolution gene expression profiles of adult mouse brains were reviewed. The LSv predominantly consisted of two neuronal populations: inhibitory GABAergic neurons and excitatory glutamatergic neurons. Approximately 90% of c‐fos‐positive cells in the LSv were identified as GABAergic neurons (Figure 2D,E). Furthermore, the number of c‐fos‐positive cells co‐expressing GABA was significantly reduced in the LSv of LPS‐treated mice but was markedly elevated after nomilin administration (Figure 2D,E). These findings suggest that nomilin exerts antidepressant effects by enhancing the excitability of GABAergic neurons in the LSv, providing mechanistic insights into its role in alleviating depressive‐like behaviors.

### 3.3 Nomilin Exerts Its Antidepressant Effects via LSv GABAergic Neurons

**FIGURE 3:**
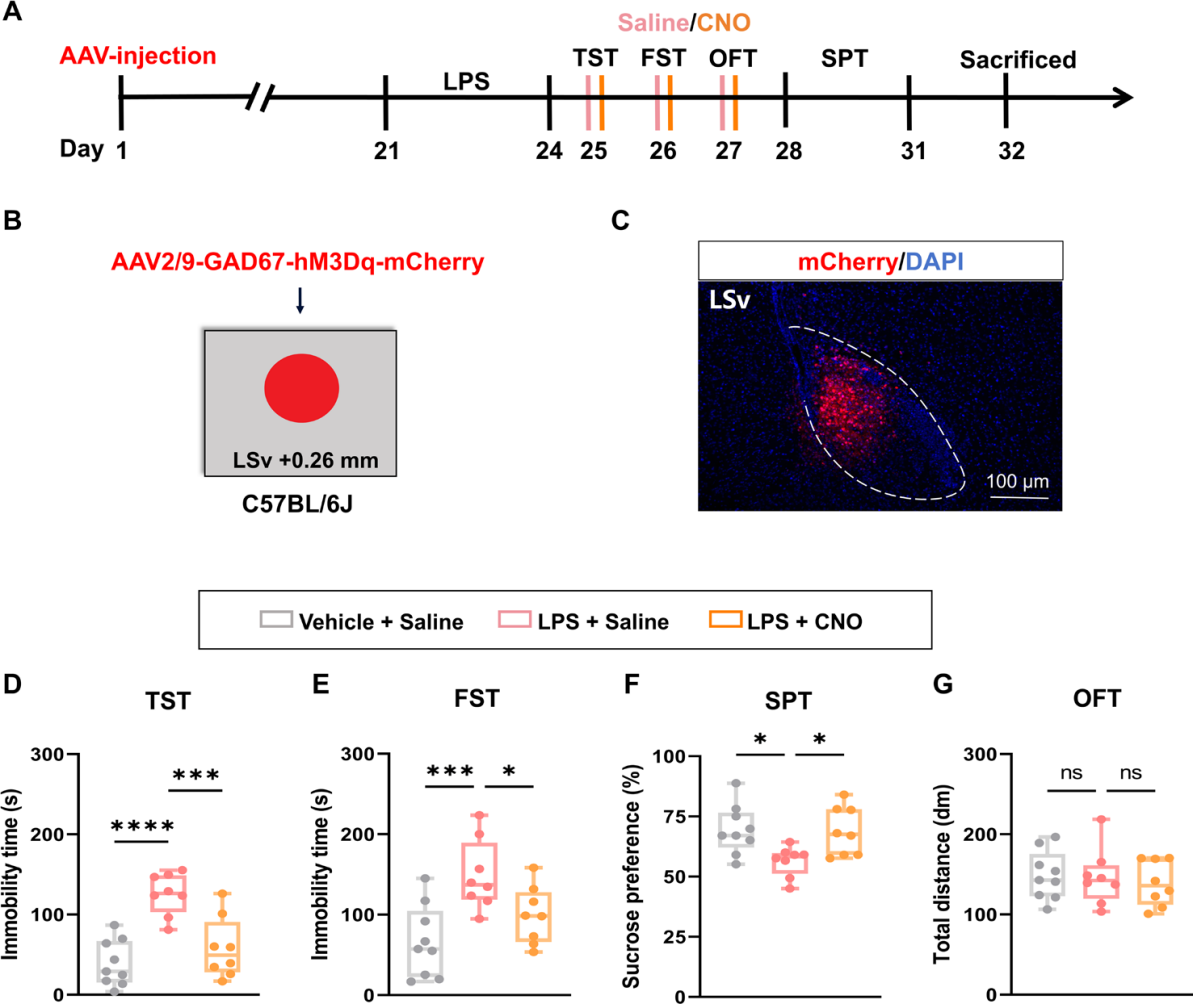
Chemogenetic activation of LSv GABAergic neurons alleviates depressive‐like behaviors in LPS‐treated mice. (A) Experimental design. (B, C) Schematic (B) and representative image (C) of viral injection site and expression in the LSv. (D) Immobility time in the TST (*n* = 8–9 mice/group; One‐way ANOVA with Dunnett's *post hoc* test, F_2, 22_ = 17.58, *p* < 0.0001). (E) Immobility time in the FST (*n* = 8–9 mice/group; One‐way ANOVA with Dunnett's *post hoc* test, F_2, 22_ = 8.436, *p* = 0.0019). (F) Sucrose preference in the SPT (*n* = 8–9 mice/group; One‐way ANOVA with Dunnett's *post hoc* test, F_2, 22_ = 5.372, *p* = 0.0126). (G) Total distance traveled in the OFT (*n* = 8–9 mice/group; One‐way ANOVA with Dunnett's *post hoc* test, F_2, 22_ = 0.2038, *p* = 0.8171). Boxplot data are presented as median, quartile, and extremum values. Significance levels ns *p* > 0.05; ^*^ *p* < 0.05; ^**^ *p* < 0.01; ^***^ *p* < 0.001, ^****^ *p* < 0.0001.

To determine whether direct activation of GABAergic neurons in the LSv can mitigate depressive‐like behaviors in mice, an AAV vector carrying hM3Dq and red fluorescent protein under the control of a GABAergic neuron‐specific promoter (AAV2/9‐GAD67‐hM3Dq‐mCherry) was bilaterally infused into the LSv. At 21 days post‐injection, depressive‐like behaviors were induced by LPS administration, followed by behavioral testing conducted after administering CNO or saline (Figure 3A,B). Proper localization of viral injections into the LSv was confirmed (Figure 3C). C‐fos staining results showed that CNO injection significantly increased the activity of GABAergic neurons in LSv (Figure S7A). The activation of LSv GABAergic neurons reversed LPS‐induced depressive‐like behaviors, as evidenced by reduced immobility in the TST (Figure 3D) and FST (Figure 3E), as well as increased sucrose consumption in the SPT (Figure 3F and Figure S1E). Notably, this activation did not affect locomotor activity, as shown by unchanged total distance traveled in the OFT (Figure 3G). To sum up, these results demonstrate that specific activation of LSv GABAergic neurons effectively alleviates LPS‐induced depressive‐like behaviors in mice.

**FIGURE 4:**
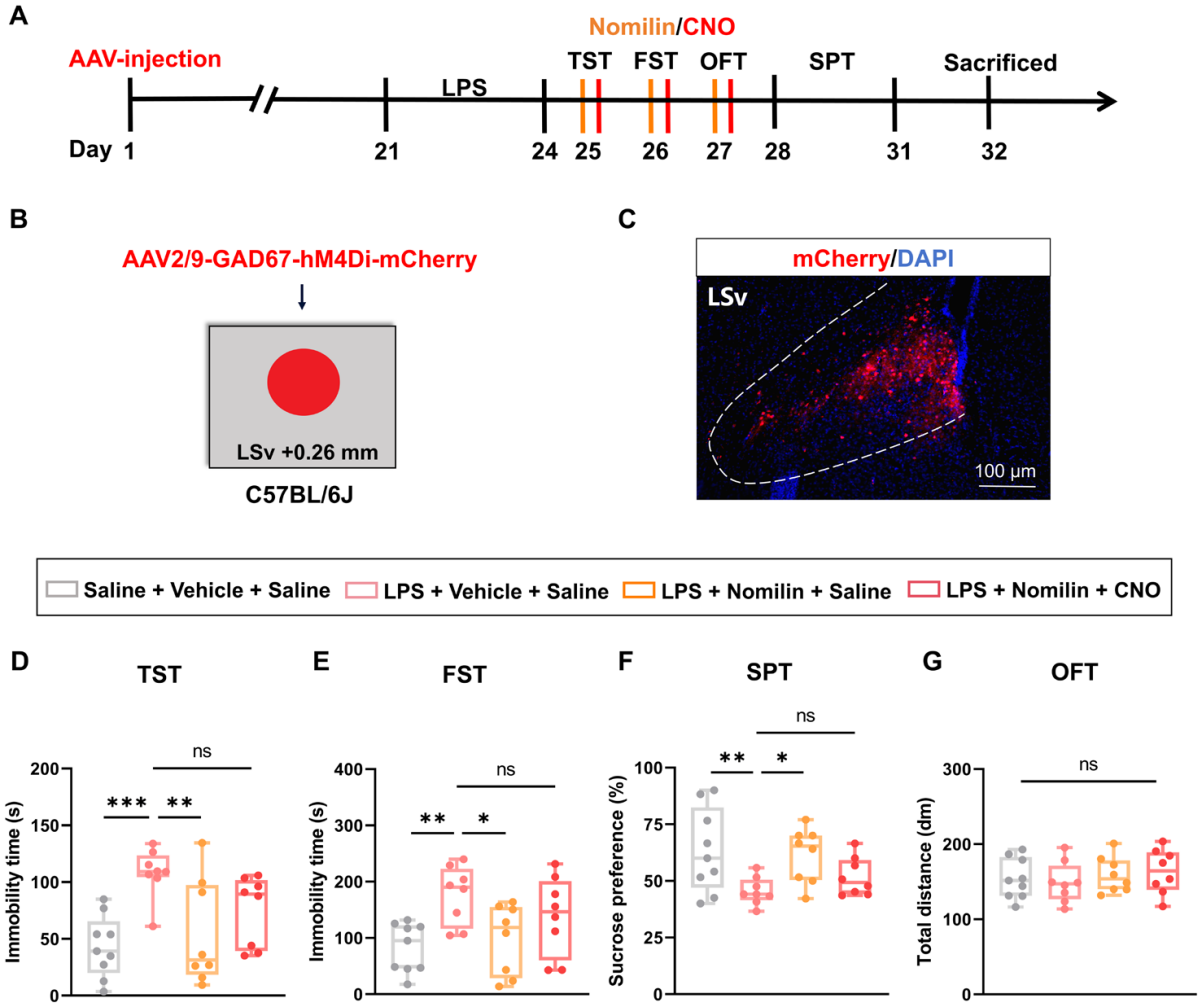
Chemogenetic inhibition of LSv GABAergic neurons in LPS‐treated mice attenuates the antidepressant effects of nomilin. (A) Experimental design. (B, C) Schematic (B) and representative image (C) of viral injection site and expression in the LSv. (D) Immobility time in the TST (*n* = 8–9 mice/group; Two‐way ANOVA with Fisher's LSD test; Main effect of nomilin: F_1, 29_ = 0.8595, *p* = 0.3615; Main effect of CNO: F_1, 29_ = 14.01, *p* = 0.0008; Interaction: F_1, 29_ = 3.883, *p* = 0.0584). (E) Immobility time in the FST (*n* = 8–9 mice/group; Two‐way ANOVA with Fisher's LSD test; Main effect of nomilin: F_1, 29_ = 0.3209, *p* = 0.5754; Main effect of CNO: F_1, 29_ = 11.19, *p* = 0.0023; Interaction: F_1, 29_ = 1.747, *p* = 0.1965). (F) Sucrose preference in the SPT (*n* = 8–9 mice/group; Two‐way ANOVA with Fisher's LSD test; Main effect of nomilin: F_1, 29_ = 0.2665, *p* = 0.6096; Main effect of CNO: F_1, 29_ = 9.962, *p* = 0.0037; Interaction: F_1, 29_ = 0.8925, *p* = 0.3526). (G) Total distance traveled in the OFT (*n* = 8–9 mice/group; Two‐way ANOVA with Bonferroni's *post hoc* multiple comparisons test; Main effect of nomilin: F_1, 29_ = 1.119, *p* = 0.2988; Main effect of CNO: F_1, 29_ = 0.002829, *p* = 0.9579; Interaction: F_1, 29_ = 0.2652, *p* = 0.6105). Boxplot data are presented as median, quartile, and extremum values. Significance levels: Ns *p* > 0.05; ^*^ *p* < 0.05; ^**^ *p* < 0.01; ^***^ *p* < 0.001.

To further investigate the role of LSv GABAergic neuronal activity in the antidepressant effects of nomilin, an AAV vector carrying hM4Di and mCherry under the control of a GABAergic neuron‐specific promoter (AAV2/9‐GAD67‐hM4Di‐mCherry) was bilaterally infused into the LSv to suppress GABAergic neuronal excitability. At 3 weeks post‐injection, nomilin or vehicle was administered to LPS‐treated mice 1 h before behavioral testing, followed by CNO to suppress LSv GABAergic neuronal activity (Figures 4A–C and S7B). Nomilin treatment effectively alleviated depressive‐like behaviors in LPS‐treated mice. However, inhibition of GABAergic neurons in the LSv significantly weakened the antidepressant effects of nomilin, as indicated by increased immobility in the TST (Figure 4D) and FST (Figure 4E), along with reduced sucrose preference in the SPT (Figure 4F and Figure S1F). Locomotor activity remained unaffected, as shown by the unchanged OFT results (Figure 4G). Collectively, these data reveal that inhibiting LSv GABAergic neurons attenuates the antidepressant efficacy of nomilin, underscoring the critical role of these neurons in its therapeutic action.

### 3.4 Nomilin Enhances Calcium Activity of LSv to BNST GABAergic Projections

To investigate the neural circuits engaged by LSv GABAergic neurons in the antidepressant effects of nomilin, projection patterns were examined using AAV2/9‐GAD67‐mCherry. LSv^GABA^ neurons were found to project to several downstream regions, including mood‐related regions such as the BNST, LHA, lateral habenula (LHb), and paraventricular nucleus (PVN) (Figure S8A). Notably, c‐fos expression was significantly elevated in the BNST of LPS‐treated mice but normalized following nomilin treatment, whereas no significant changes were observed in other regions (Figure S8B). These findings suggest that BNST serves as a primary downstream target of LSv GABAergic neurons, contributing to the antidepressant effects of nomilin.

**FIGURE 5:**
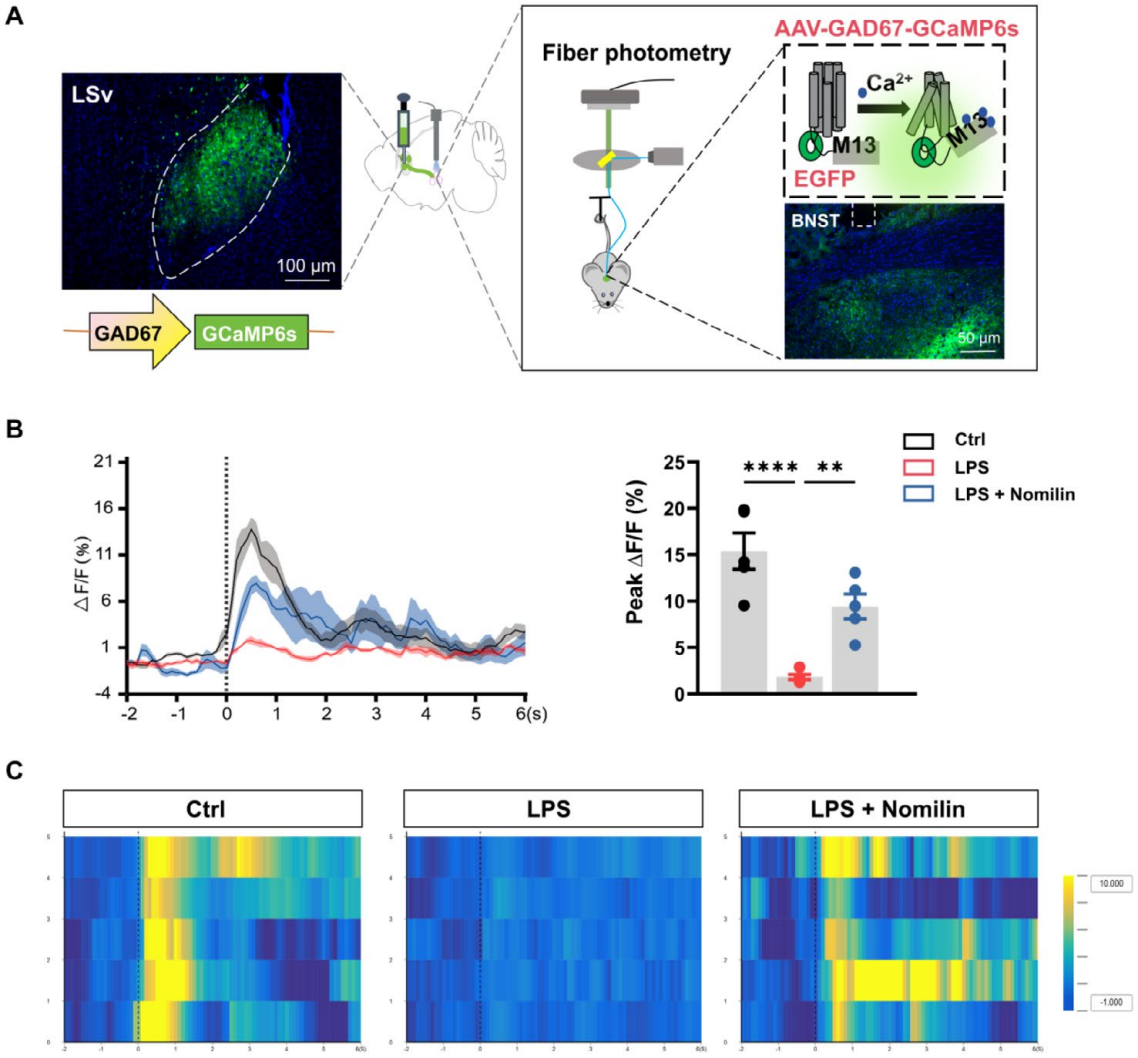
Nomilin enhances calcium activity in LSv to BNST GABAergic projections. (A) Schematic and representative images of viral injection site in the LSv and cannula site in the BNST. (B) Representative traces and quantitative analysis of normalized Ca^2+^ fluorescence (ΔF/F) (*n* = 5 mice/group; One‐way ANOVA with Dunnett's *post hoc* test, F_2, 12_ = 24.46, *p* < 0.0001). (C) Representative heatmaps of normalized Ca^2+^ fluorescence (ΔF/F). Data are presented as mean ± SEM. Significance level: ^**^ *p* < 0.01.

To confirm whether the LSv^GABA^ → BNST neural circuit is activated by nomilin, calcium activity within this pathway was measured using fiber photometry during the TST, 1 h after administration. AAV‐GAD67‐GCaMP6s‐EGFP was infused into the LSv, and optical fibers were implanted into the BNST to record calcium transients (Figure 5A). Nomilin‐treated mice exhibited a significant increase in calcium signals in BNST‐projecting LSv GABA neurons, effectively reversing the suppressed calcium activity observed in LPS‐treated mice (Figure 5B,C). These results demonstrate that nomilin enhances the excitability of the LSv^GABA^ → BNST neural circuit, underscoring its role in alleviating depressive‐like behaviors in mice.

### 3.5 LSv^GABA^ → BNST Neural Circuit Mediates the Antidepressant Effects of Nomilin

**FIGURE 6:**
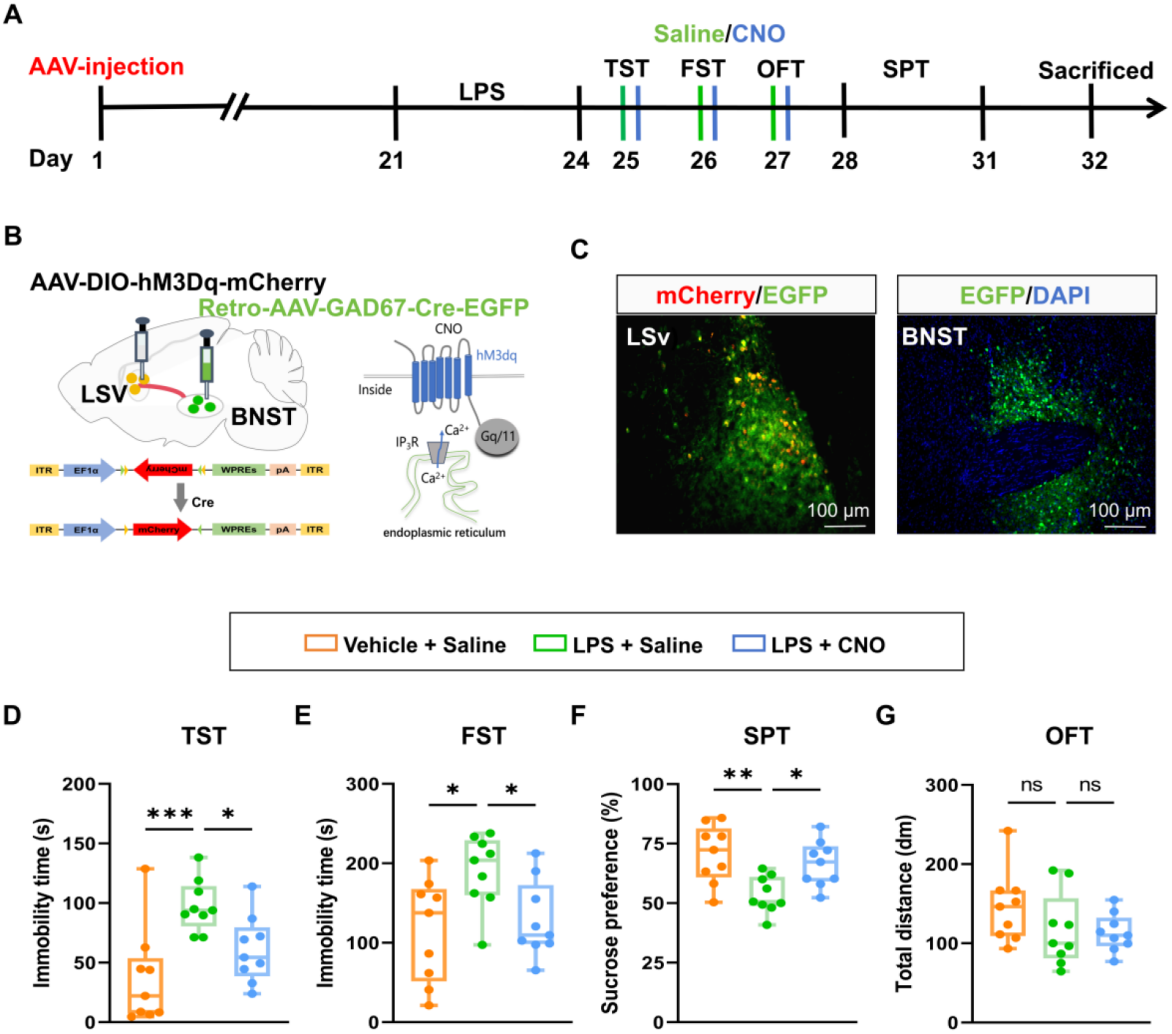
Chemogenetic activation of the LSv^GABA^ → BNST neural circuit alleviates depressive‐like behaviors in LPS‐treated mice. (A) Experimental design. (B, C) Schematic (B) and representative images (C) of viral injection site and expression in the LSv and BNST. (D) Immobility time in the TST (*n* = 9 mice/group; One‐way ANOVA with Dunnett's *post hoc* test, F_2, 24_ = 8.801, *p* = 0.0014). (E) Immobility time in the FST (*n* = 9 mice/group; One‐way ANOVA with Dunnett's *post hoc* test, F_2, 24_ = 5.003, *p* = 0.0153). (F) Sucrose preference in the SPT (*n* = 9 mice/group; One‐way ANOVA with Dunnett's *post hoc* test, F_2, 24_ = 7.291, *p* = 0.0034). (G) Total distance traveled in the OFT (*n* = 9 mice/group; One‐way ANOVA with Dunnett's *post hoc* test, F_2, 24_ = 1.783, *p* = 0.1896). Boxplot data are presented as median, quartile, and extremum values. Significance levels: Ns *p* > 0.05; ^*^ *p* < 0.05; ^**^ *p* < 0.01; ^***^ *p* < 0.001.

To assess whether the LSv^GABA^ → BNST neural circuit contributes to the modulation of depression‐like behaviors induced by LPS, AAV‐DIO‐hM3Dq‐mCherry was bilaterally injected into the LSv to enable chemogenetic activation of this pathway. Retro‐AAV‐GAD67‐Cre‐EGFP was infused into the BNST to label GABAergic neurons projecting to this region (Figure 6A,B). Immunofluorescence revealed mCherry‐positive neuronal bodies and terminals in the LSv, along with EGFP‐positive neuronal bodies in the BNST, confirming the targeted manipulation of this circuit (Figure 6C). Behavioral analyses demonstrated that the activation of BNST‐projecting LSv GABAergic neurons by CNO significantly alleviated LPS‐induced depressive‐like behaviors. Improvements were observed in the TST (Figure 6D), FST (Figure 6E), and SPT (Figures 6F and S1G), while locomotor activity, as assessed by total distance traveled in the OFT (Figure 6G), remained unaffected. These findings indicate that activation of the LSv^GABA^ → BNST neural circuit effectively alleviates depressive‐like behaviors in LPS‐treated mice.

**FIGURE 7:**
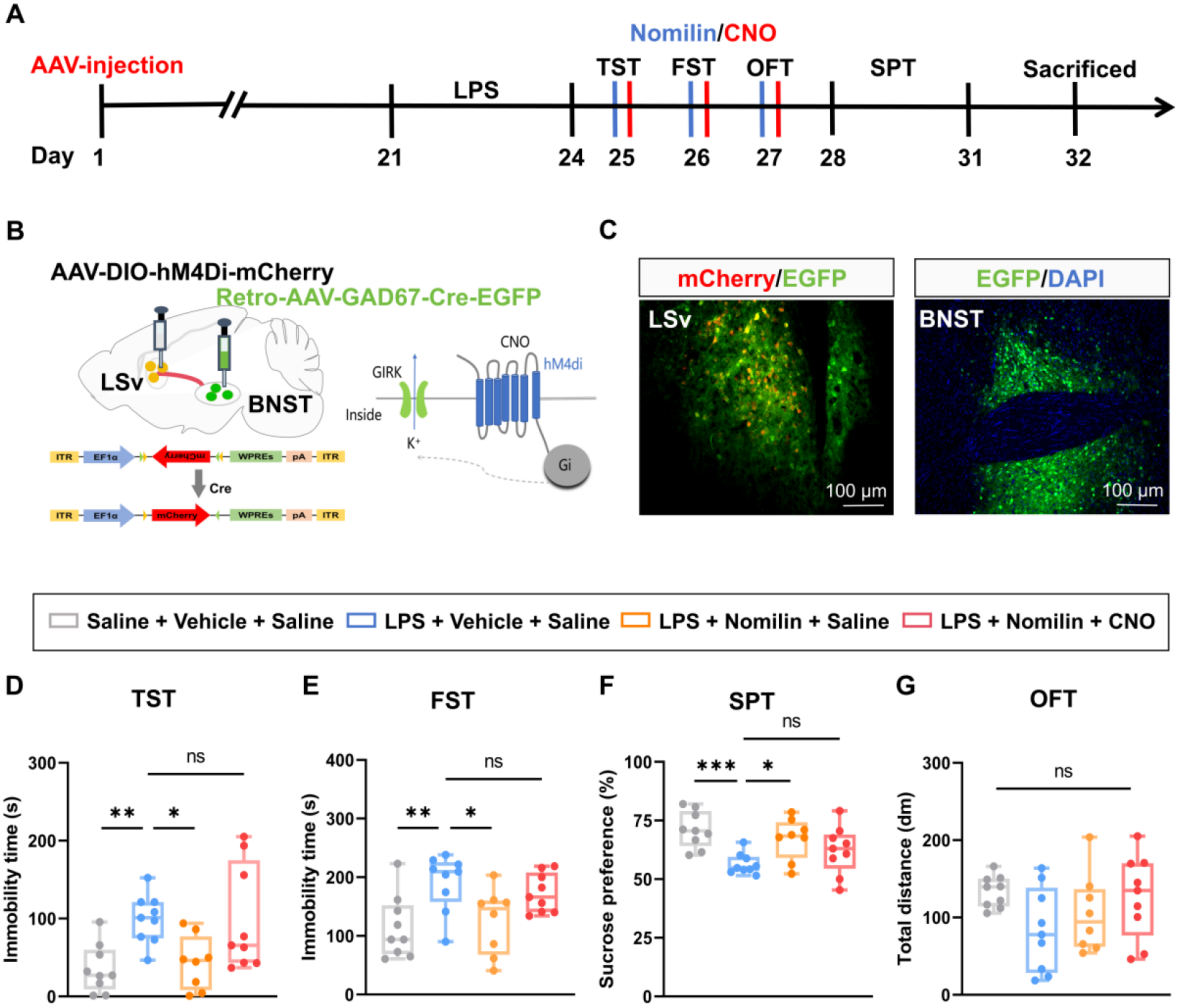
Chemogenetic inhibition of the LSv^GABA^ → BNST circuit in LPS‐treated mice attenuates the antidepressant effect of nomilin. (A) Experimental design. (B, C) Schematic (B) and representative images (C) of viral injection site and expression in the LSv and BNST. (D) Immobility time in the TST (*n* = 8–9 mice/group; Two‐way ANOVA with Fisher's LSD test; Main effect of nomilin: F_1, 31_ = 0.04310, *p* = 0.8369; Main effect of CNO: F_1, 31_ = 15.91, *p* = 0.0004; Interaction: F_1, 31_ = 0.09622, *p* = 0.7585). (E) Immobility time in the FST (*n* = 8–9 mice/group; Two‐way ANOVA with Fisher's LSD test; Main effect of nomilin: F_1, 31_ = 0.05827, *p* = 0.8108; Main effect of CNO: F_1, 31_ = 14.45, *p* = 0.0006; Interaction: F_1, 31_ = 0.8064, *p* = 0.3761). (F) Sucrose preference in the SPT (*n* = 8–9 mice/group; Two‐way ANOVA with Fisher's LSD test; Main effect of nomilin: F_1, 31_ = 0.1213, *p* = 0.7300; Main effect of CNO: F_1, 31_ = 12.09, *p* = 0.0015; Interaction: F_1, 31_ = 2.788, *p* = 0.1051). (G) Total distance traveled in the OFT (*n* = 8–9 mice/group; Two‐way ANOVA with Bonferroni's *post hoc* multiple comparisons test; Main effect of nomilin: F_1, 31_ = 0.1988, *p* = 0.6580; Main effect of CNO: F_1, 31_ = 0.7807, *p* = 0.3837; Interaction: F_1, 31_ = 5.090, *p* = 0.0313). Boxplot data are presented as median, quartile, and extremum values. Significance levels: Ns *p* > 0.05; ^*^ *p* < 0.05; ^**^ *p* < 0.01.

To further investigate the role of this neural circuit in the antidepressant effects of nomilin, chemogenetic inhibition of the LSv^GABA^ → BNST pathway was achieved by infusing AAV‐DIO‐hM4Di‐mCherry into the LSv and Retro‐AAV‐GAD67‐Cre‐EGFP into the BNST (Figures 7A and 6B). Immunofluorescence validated successful viral expression (Figure 7C). Behavioral tests revealed that nomilin treatment effectively rescued depressive‐like behaviors in LPS mice, as evidenced by improved performance in the TST (Figure 7D), FST (Figure 7E), and SPT (Figure 7F and Figure S1H). However, chemogenetic inhibition of the LSv^GABA^ → BNST circuit significantly attenuated these effects, leading to a reappearance of depressive‐like behaviors. Locomotor activity, as assessed by the OFT (Figure 7G), showed no significant changes between groups. These findings demonstrate that the LSv^GABA^ → BNST neural circuit is critical for the antidepressant effects of nomilin.

### 3.6 GABA_A_ Receptors Mediate the LSv^GABA^ → BNST Neural Circuit to Regulate Depressive‐Like Behaviors in Mice

**FIGURE 8:**
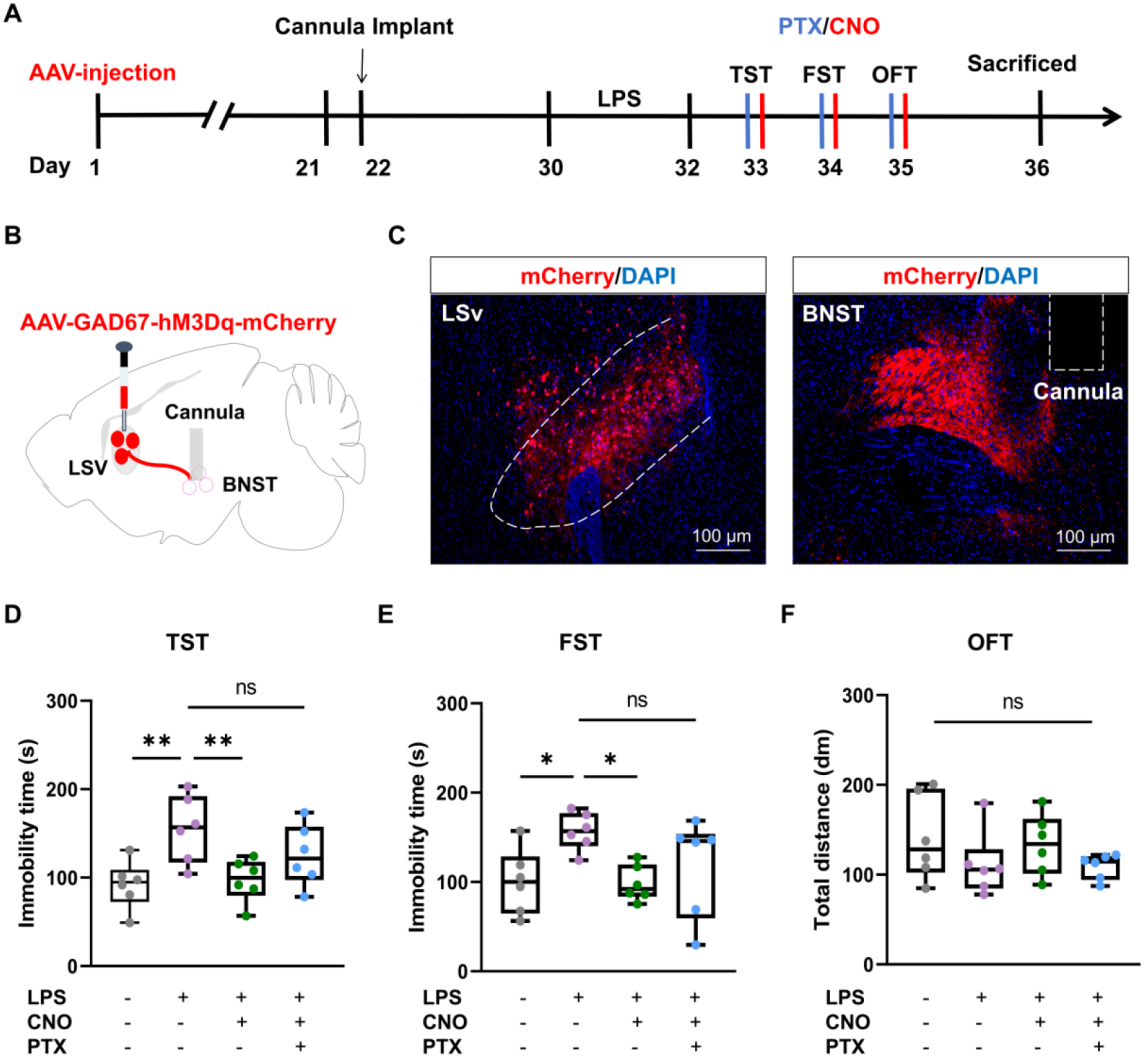
GABA_A_ receptors mediate the LSv^GABA^ → BNST neural circuit to regulate depressive‐like behaviors in mice. (A) Experimental design. (B) Schematic of viral infusion in the LSv and cannula implantation in the BNST. (C) Representative images of viral expression in the LSv and cannula site in the BNST. (D) Immobility time in the TST (*n* = 6 mice/group; Two‐way ANOVA with Fisher's LSD test; Main effect of nomilin: F_1, 20_ = 0.9186, *p* = 0.3493; Main effect of CNO: F_1, 20_ = 12.59, *p* = 0.0020; Interaction: F_1, 20_ = 1.898, *p* = 0.1836). (E) Immobility time in the FST (*n* = 6 mice/group; Two‐way ANOVA with Fisher's LSD test; Main effect of nomilin: F_1, 20_ = 1.786, *p* = 0.1964; Main effect of CNO: F_1, 20_ = 6.915, *p* = 0.0161; Interaction: F_1, 20_ = 1.683, *p* = 0.2093). (F) Total distance traveled in the OFT (*n* = 6 mice/group; Two‐way ANOVA with Tukey's *post hoc* multiple comparisons test; Main effect of nomilin: F_1, 20_ = 0.1120, *p* = 0.7413; Main effect of CNO: F_1, 20_ = 3.544, *p* = 0.0744; Interaction: F_1,20_ = 0.03620, *p* = 0.8510). Boxplot data are presented as median, quartile, and extremum values. Significance levels: Ns *p* > 0.05; ^*^ *p* < 0.05; ^**^ *p* < 0.01.

Next, the type of receptors in the BNST involved in the regulation of LSv^GABA^ → BNST neural circuit was further elucidated. Three weeks after AAV2/9‐GAD67‐hM3Dq‐mCherry infusion in the LSv, cannulas were implanted into the BNST to allow precise delivery of the GABA_A_ receptor antagonist PTX (Figure 8A–C). As expected, activation of LSv^GABA^ → BNST circuit with CNO significantly alleviated depressive‐like behaviors in LPS‐treated mice. In contrast, the administration of PTX into the BNST reversed these antidepressant effects, as demonstrated by the increased immobility in the TST (Figure 8D) and FST (Figure 8E). Notably, PTX infusion did not affect locomotor activity, as indicated by unchanged OFT results (Figure 8F). To conclude, these findings reveal that GABA_A_ receptors in the BNST are critical mediators of the antidepressant effects of the LSv^GABA^ → BNST neural circuit, underscoring their role in regulating depressive‐like behaviors in mice.

## 4 Discussion

This study provides the first evidence of the distinct antidepressant effects of nomilin in modulating depressive‐like behaviors in mice. Notably, nomilin administration significantly activated LSv GABAergic neurons and the LSv^GABA^ → BNST neural circuit in LPS‐treated mice, which played a key role in its antidepressant mechanism. Furthermore, GABA_A_ receptors in the BNST were identified as components of the LSv^GABA^ → BNST circuit involved in the regulation of depressive‐like behaviors in mice.

Although depression is widespread, its pathogenesis remains poorly understood and is believed to result from a complex interplay of genetic, environmental, and social stress‐related factors [52]. During the development of antidepressant therapies, drug‐induced and stress‐induced mouse models are commonly employed to replicate depressive‐like states. In this study, LPS‐ and CRS‐induced depressive mouse models were used to evaluate the effects of nomilin across distinct pathophysiological mechanisms. LPS‐induced models are often used to mimic immune activation‐driven depressive‐like symptoms, while CRS‐induced models simulate stress‐related depressive‐like behaviors through overactivation of the hypothalamic–pituitary–adrenergic (HPA) axis ([53, 54]; Ma et al., 2023; [55, 56, 57, 58, 59, 60, 61, 62]). This study demonstrated that a single oral dose of 20 and 40 mg/kg nomilin effectively alleviated behavioral despair and anhedonia in LPS‐treated mice. In contrast, depressive‐like behaviors in the CRS model required three consecutive days of 40 mg/kg nomilin treatment to achieve therapeutic effects. The differing response times between the models may reflect variations in the underlying mechanisms driving depressive behaviors. Behavioral assessments, including the CPP, SPT, EPM, and OFT, revealed no adverse effects of nomilin on natural reward preference, anxiety‐like behaviors, or locomotor activity in normal mice. These findings indicate a favorable safety profile for nomilin, highlighting its potential as a promising candidate for the development of innovative antidepressant therapies.

The LSv is a critical brain region involved in a range of physiological functions and behaviors, such as depression [63], anxiety [64], feeding [65], and cognition [66]. Its neuronal population is predominantly composed of inhibitory GABAergic neurons, with a smaller proportion of excitatory glutamatergic neurons [67, 68]. Recent research has reported that sucrose consumption increases c‐fos expression in LSv GABAergic neurons in chronic unpredictable mild stress model mice [69]. In addition, clinical evidence has shown reduced GABA levels in the cerebrospinal fluid and cerebral cortex of patients with depression [68]. In the present study, a significant increase in c‐fos expression was observed in LSv GABAergic neurons following nomilin administration in LPS‐treated mice. Activation of these neurons alleviated LPS‐induced depressive‐like behaviors, whereas their inhibition attenuated the antidepressant effects of nomilin. These findings underscore the critical role of LSv GABAergic neurons in mediating the antidepressant effects of nomilin.

The BNST is a critical forebrain structure essential for emotional regulation and stress responses ([70, 71, 72, 73, 74, 75, 76]). Previous research has demonstrated that various stressors enhance neuronal activity in the BNST [77], while clinical data suggest that stimulation of BNST to NAc projections can effectively improve symptoms in depression patients [78]. Interestingly, in this study, the BNST was identified as a major downstream target of LSv GABAergic projections, exhibiting increased activity in calcium signal recordings following nomilin administration. Chemogenetic modulation of the LSv^GABA^ → BNST circuit further confirmed that this pathway plays a key role in regulating depressive‐like behaviors. Despite these findings, the specific neuronal subtypes in the BNST involved in this circuit remain poorly characterized. Future research is needed to elucidate the precise mechanisms by which BNST neurons contribute to the LSv^GABA^ → BNST circuit and its role in antidepressant effects.

Synaptic transmission serves as the primary mode of communication between neurons, wherein presynaptic neurotransmitters bind to specific receptors on the postsynaptic membrane, generating either excitatory or inhibitory postsynaptic potential. In the BNST, a range of receptor types, including 5‐HT and GABA receptors, mediate such interactions [79, 80]. Given the pivotal role of GABAergic neurons in modulating LSv activity in depression‐like mice, we speculate that GABA receptors in the BNST may be involved in the antidepressant effects of the LSv^GABA^ → BNST neural circuit. GABA receptors in the brain are primarily categorized into GABA_A_ and GABA_B_ receptors [81]. Clinical studies have demonstrated that oral administration of the GABA_A_ receptor agonist zuranolone for 14 days markedly improves symptoms in individuals with major depression [82]. To investigate the involvement of GABA receptor subtypes in the LSv^GABA^ → BNST circuit, a cannula was implanted into the BNST for localized delivery of the GABA_A_ receptor antagonist PTX. Results showed that antagonism of the GABA_A_ receptor significantly diminished the antidepressant effects of the LSv^GABA^ → BNST neural circuit. These findings suggest that LSv GABAergic neurons exert their antidepressant effects by targeting BNST neurons via GABA_A_ receptors.

## 5 Conclusions

This study identified a previously unrecognized role of nomilin in alleviating depressive‐like behaviors in LPS‐treated mice. Notably, LSv GABAergic neurons participated in the antidepressant effects of nomilin via the LSv^GABA^ → BNST circuit, which may serve as a novel target for the treatment of depression. This research advances our understanding of the mechanisms underlying depression and provides a potential therapeutic strategy for the development of antidepressant drugs.

## Author Contributions

**Liang Chen, Boli Fu, and Jiaxin Liu:** investigation, data curation, formal analysis, writing, original draft. **Cun Wang and Weijun Huang:** methodology, validation. **Danhua Yuan and Chen Qing:** formal analysis, project administration. **Hao Hong and Yao Zhang:** conceptualization, writing, review and editing, supervision, Project administration, funding acquisition. All authors read and approved the manuscript.

## Ethics Statement

This study was approved by the Animal Ethics Committee of China Pharmaceutical University (Approval No. 2023–08–023).

## Conflicts of Interest

The authors declare no conflicts of interest.

## Supporting information


**Data S1:** Supporting Information.

## Data Availability

The data that support the findings of this study are available from the corresponding author upon reasonable request.
