# Supplementary material for: Nomilin Regulates Depressive‐Like Behaviors in Mice via the Ventral Part of the Lateral Septum to Bed Nucleus of the Stria Terminalis Circuit
Source: CNS Neurosci Ther. 2025 Nov 24;31(11):e70647. doi: 10.1111/cns.70647 (PMC12644931; doi:10.1111/cns.70647)
Supplement: Supplementary file 1 — Data S1: Supporting Information. [file CNS-31-e70647-s001.zip › cns70647-sup-0010-FigureS1-S8@Supplementary Materials - 1.docx]

**Nomilin regulates depressive-like behaviors in mice via the ventral part of the lateral septum to bed nucleus of the stria terminalis circuit**

***Supplemental Materials***

**Supplemental Methods**

1. **Enzyme-Linked Immunosorbent Assay (ELISA)**

Brain tissues in each group were homogenized in RIPA buffer (Thermo Fisher Scientific, USA) containing PMSF (Bio-Rad, USA) at a general weight/volume (mg/μL) ratio of 1:10 using a homogenizer on ice. The homogenates were centrifuged at 12,000 rpm for 10 min at 4 °C, and the supernatant was collected to represent extracellular components. BCA Protein Assay Kit (Beyotime, China) was used to measure the total extracted protein from brain samples according to manufacturer's instructions. Cytokine levels were measured according to the instructions provided with ELISA kits for IL-6, IL-1β, and TNF-α (Lapuda, China). Each sample was measured at 450-nm wavelength using a microplate reader (VLBL00D0, Thermo Fisher Scientific, USA).

1. **LC-MS/MS Measurement of Nomilin Concentration in Brain Tissue**

Male mice were anesthetized with isoflurane and decapitated 60 minutes after oral administration of nomilin. Brains were immediately excised on an ice-cooled platform, then rapidly frozen in liquid nitrogen and stored at -80 °C until analysis. Upon thawing, brain tissues were homogenized in ice-cold ddH2O (150 μL per 2 mg tissue) using a cryogenic grinder to prepare tissue homogenates. Standard calibration curves were generated by spiking 2.5 μL of nomilin at varying concentrations (0.025, 0.25, 0.5, 1, 2.5, 5, 10 and 20 pg/mL) with 47.5 μL of drug-naive brain tissue homogenate. 200 μL of brain homogenate was mixed with 800 μL of cold methanol-acetonitrile (1:3, v/v) solution. After vortexing for 1 min and incubation at 4 °C for 10 min, samples were centrifuged at 16,000 g for 10 min at 4 °C. The supernatant was collected, and the precipitation procedure was repeated. Combined supernatants were used for LC-MS/MS analysis. Nomilin concentrations were determined by LC-MS/MS with modifications to previously described methods. The analytical system consisted of: Column: Acquity UPLC HSS T3 C18 .Mobile phase: A: 0.1% formic acid in water, B: Acetonitrile, Flow rate: 0.3 mL/min, Column temperature: 40 °C, Injection volume: 1 μL, Gradient Program: 0-3 min: 90%A→10% B, 3-6 min: 90%A→10% B, 6-14 min: 20%A→80% B, 14-22.5 min: 5%A→95% B, 22.5-34.5 min: 5%A→95% B, 34.5-45.6 min: 90%A→10% B, 45.6-60.6 min: 90%A→10% B. Instruments: LC-30A system (Shimadzu, Japan), LCMS 8060NX triple quadrupole mass spectrometer (Shimadzu, Japan). Ionization mode: Positive electrospray ionization (ESI^+^). MRM transitions: 515.3→411.3, 515.3→161.1, 515→469.

**Supplemental Figures**

**
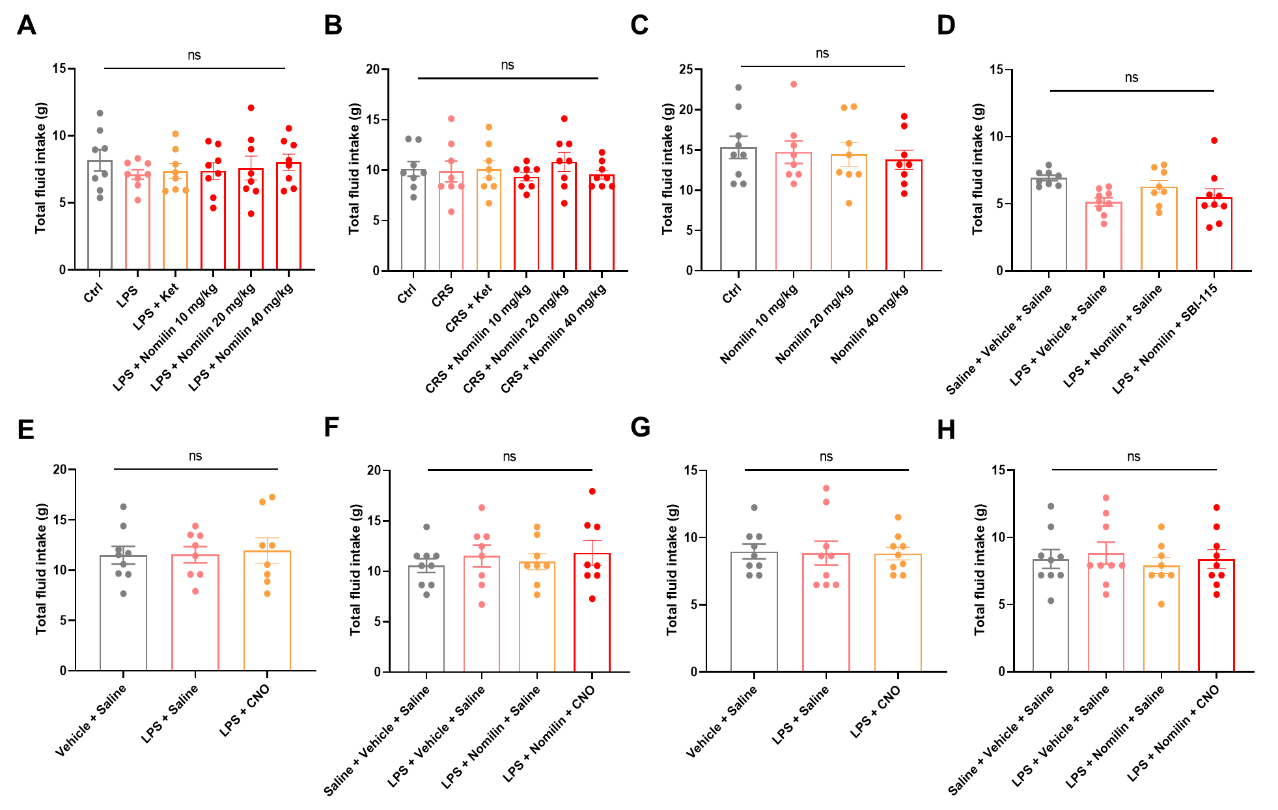
Figure S1. The total fluid intake of mice in each group remained unchanged in SPT.**

1. Total fluid intake in the SPT (n = 8 mice/group; One-way ANOVA with Dunnett’s *post-hoc* test, F_5, 42_ = 0.3993, *P* = 0.8465; related to Figure 1).
2. Total fluid intake in the SPT (n = 8 mice/group; One-way ANOVA with Dunnett’s *post-hoc* test, F_5, 42_ = 0.4415, *P* = 0.8170; related to Figure S1).
3. Total fluid intake in the SPT (n = 8–9 mice/group; One-way ANOVA with Dunnett’s *post-hoc* test, F_3, 29_ = 0.2221, *P* = 0.8803; related to Figure S2).
4. Total fluid intake in the SPT (n = 8–9 mice/group; Two-way ANOVA with Bonferroni's *post hoc* multiple comparisons test; Main effect of nomilin: F_1, 30_ = 0.1227, *P* = 0.7286; Main effect of SBI-115: F_1, 30_ = 8.243, *P* = 0.0074; Interaction: F_1, 30_ = 1.201, *P* = 0.2819; related to Figure S3).
5. Total fluid intake in the SPT (n = 8–9 mice/group; One-way ANOVA with Dunnett’s *post-hoc* test, F_2, 22_ = 0.06622, *P* = 0.9361; related to Figure 3).
6. Total fluid intake in the SPT (n = 8–9 mice/group; Two-way ANOVA with Bonferroni's *post hoc* multiple comparisons test; Main effect of nomilin: F_1, 29_ = 0.1279, *P* = 0.7233; Main effect of CNO: F_1, 29_ = 0.9133, *P* = 0.3471; Interaction: F_1, 29_ = 0.001841, *P* = 0.9661; related to Figure 4).
7. Total fluid intake in the SPT (n = 9 mice/group; One-way ANOVA with Dunnett’s *post-hoc* test, F_2, 24_ = 0.01399, *P* = 0.9861; related to Figure 6).
8. Total fluid intake in the SPT (n = 8–9 mice/group; Two-way ANOVA with Bonferroni's *post hoc* multiple comparisons test; Main effect of nomilin: F_1, 31_ = 0.3997, *P* = 0.5319; Main effect of CNO: F_1, 31_ = 0.4076, *P* = 0.5279; Interaction: F_1, 31_ = 0.0009109, *P* = 0.9761; related to Figure 7).

Data are presented as mean ± SEM. Significance levels: ns *P* > 0.05.


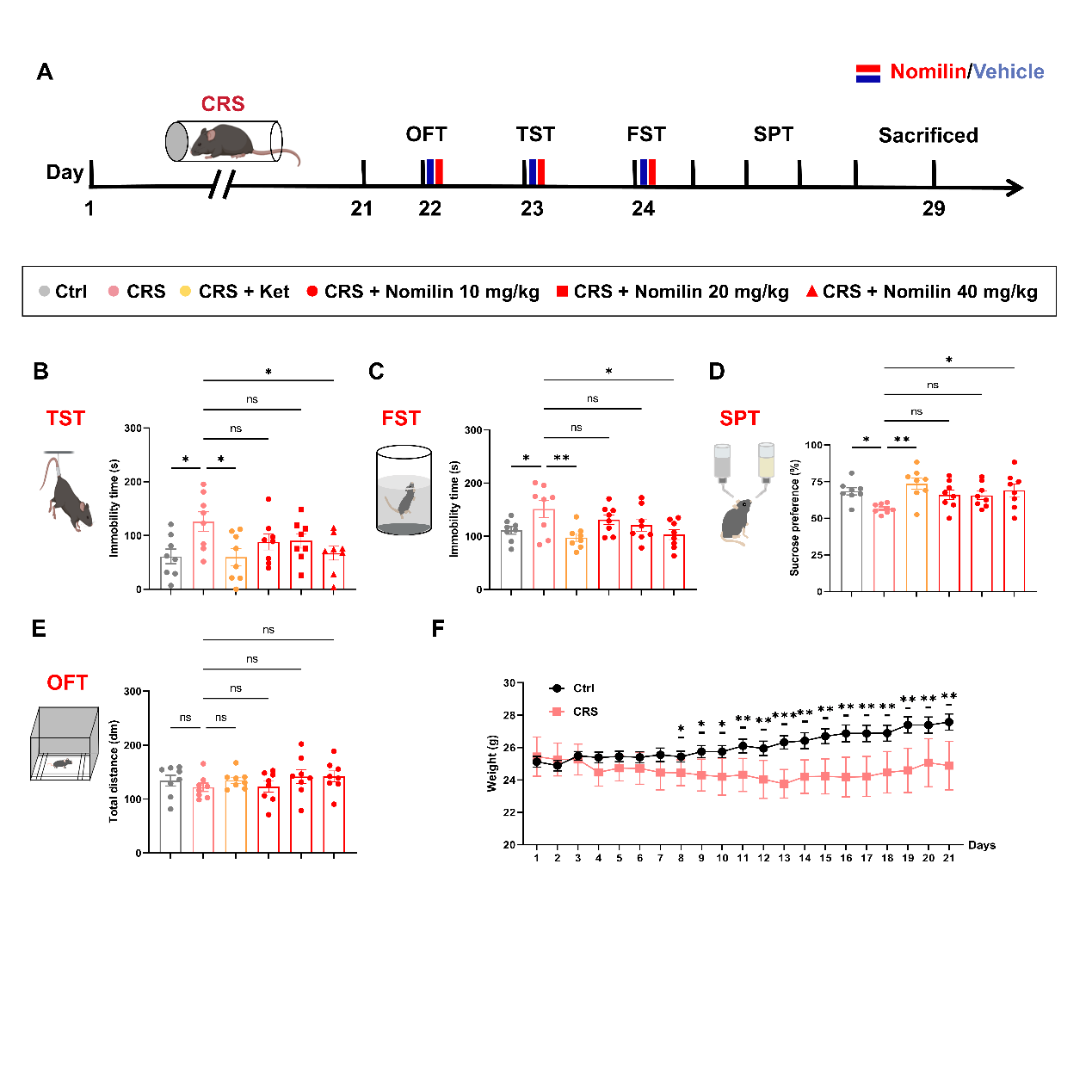


**Figure S2. Nomilin ameliorates CRS-induced depressive-like behaviors in mice.**

1. Experimental design.

(B-C) Immobility time in the TST (B) and FST (C) (n = 8 mice/group; One-way ANOVA with Dunnett’s *post-hoc* test; F_5, 42_ = 2.896, *P* = 0.0247 for TST; F_5, 42_ = 3.538, *P* = 0.0093 for FST).

(D) Sucrose preference in the SPT (n = 8 mice/group; One-way ANOVA with Dunnett’s *post-hoc* test, F_5, 42_ = 3.208, *P* = 0.0153).

(E) Total distance traveled in the OFT (n = 8 mice/group; One-way ANOVA with Dunnett’s *post-hoc* test, F_5, 42_ = 0.8237, *P* = 0.5399).

(F) Body weight measurements of mice (n = 8 mice/group; Two-tailed unpaired Student’s *t*-test; t (14) = 2.292, *P* = 0.0379 for Day 8; t (14) = 2.852, *P* = 0.0128 for Day 9; t (14) = 2.830, *P* = 0.0134 for Day 10; t (14) = 3.206, *P* = 0.0063 for Day 11; t (14) = 3.167, *P* = 0.0069 for Day 12; t (14) = 4.939, *P* = 0.0002 for Day 13; t (14) = 3.676, *P* = 0.0025 for Day 14; t (14) = 4.103, *P* = 0.0011 for Day 15; t (14) = 4.013, *P* = 0.0013 for Day 16; t (14) = 3.999, *P* = 0.0013 for Day 17; t (14) = 3.610, *P* = 0.0028 for Day 18; t (14) = 4.066, *P* = 0.0012 for Day 19; t (14) = 3.230, *P* = 0.0060 for Day 20; t (14) = 3.709, *P* = 0.0023 for Day 21).

Data are presented as mean ± SEM. Significance levels: ns *P* > 0.05; *^∗^P* < 0.05; *^∗∗^P* < 0.01; *^∗∗∗^P* < 0.001.

CRS, chronic restraint stress.


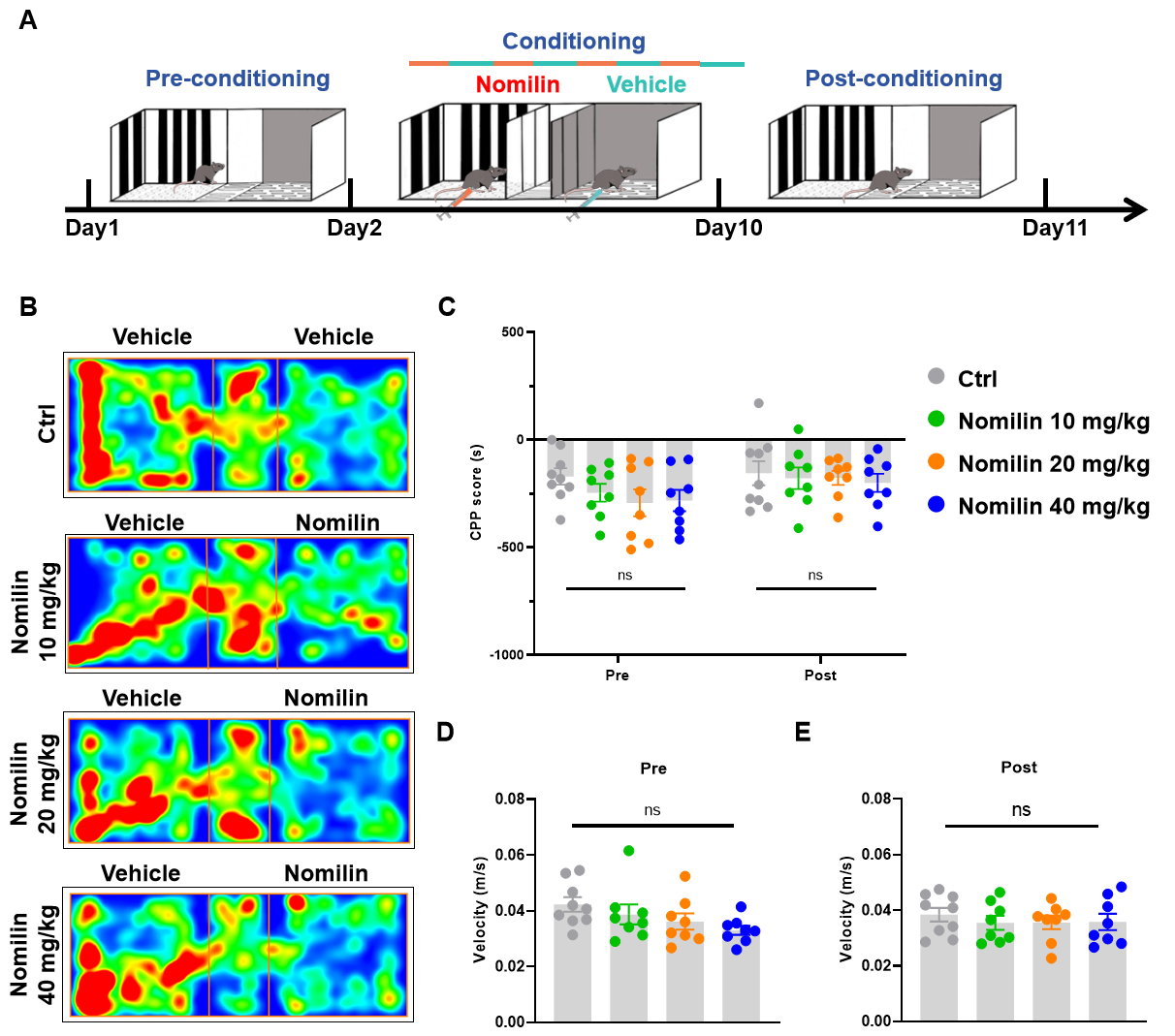

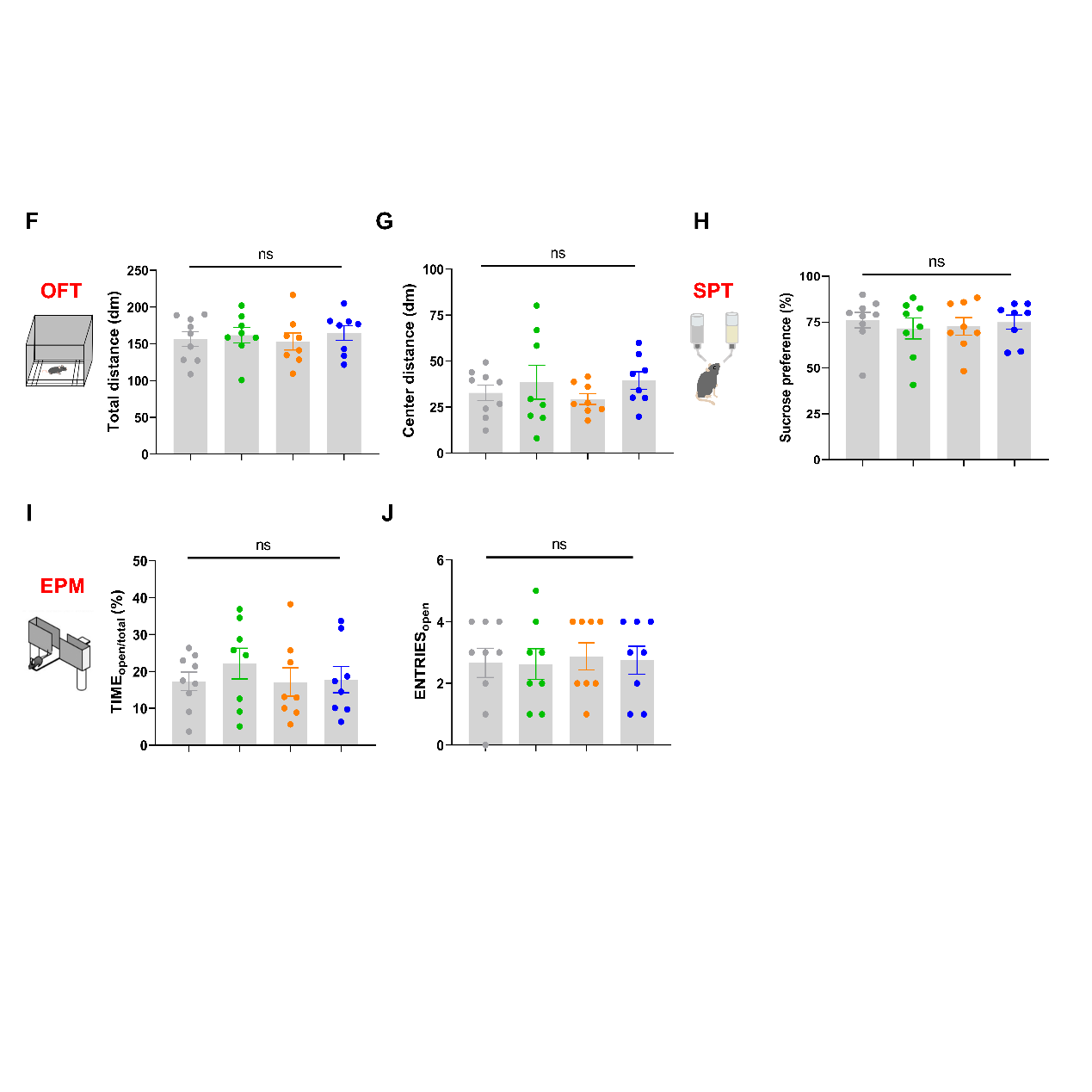


**Figure S3. Nomilin exerts no influence on natural reward preference, locomotor activity, or anxiety-like behaviors in mice.**

(A) Experimental design of CPP.

(B) Representative heatmaps of CPP.

(C) CPP scores (n = 8–9 mice/group; One-way ANOVA with Dunnett’s *post-hoc* test; F_3, 29_ = 1.389, *P* = 0.2658 for Pre; F_3, 29_ = 0.1549, *P* = 0.9257 for Post).

(D-E) Velocity in pre-conditioning (D) and post-conditioning (E) of CPP (n = 8–9 mice/group; One-way ANOVA with Dunnett’s *post-hoc* test; F_3,_ _29_ = 2.079, *P* = 0.1247 for Pre; F_3, 29_ = 0.3059, *P* = 0.8209 for Post).

(F-G) Total distance (F) and center distance (G) in OFT (n = 8–9 mice/group; One-way ANOVA with Dunnett’s *post-hoc* test; F_3, 29_ = 0.2269, *P* = 0.8769 for total; F_3, 29_ = 0.2269, *P* = 0.8769 for center).

(H) Sucrose preference in SPT (n = 8–9 mice/group; Kruskal-Wallis test with Dunn's multiple comparisons test, *P* = 0.9440).

(I-J) Time in open arms (I) and open arms entries (J) in EPM (n = 8–9 mice/group; One-way ANOVA with Dunnett’s *post-hoc* test, F_3, 29_ = 0.4457, *P* = 0.7222 for time; Brown-Forsythe and Welch ANOVA tests, F_3.000, 28.75_ = 0.05494, *P* = 0.9827 for entries).

Data are presented as mean ± SEM. Significance level: ns *P* > 0.05.

CPP, conditioned place preference; EPM, elevated plus maze.


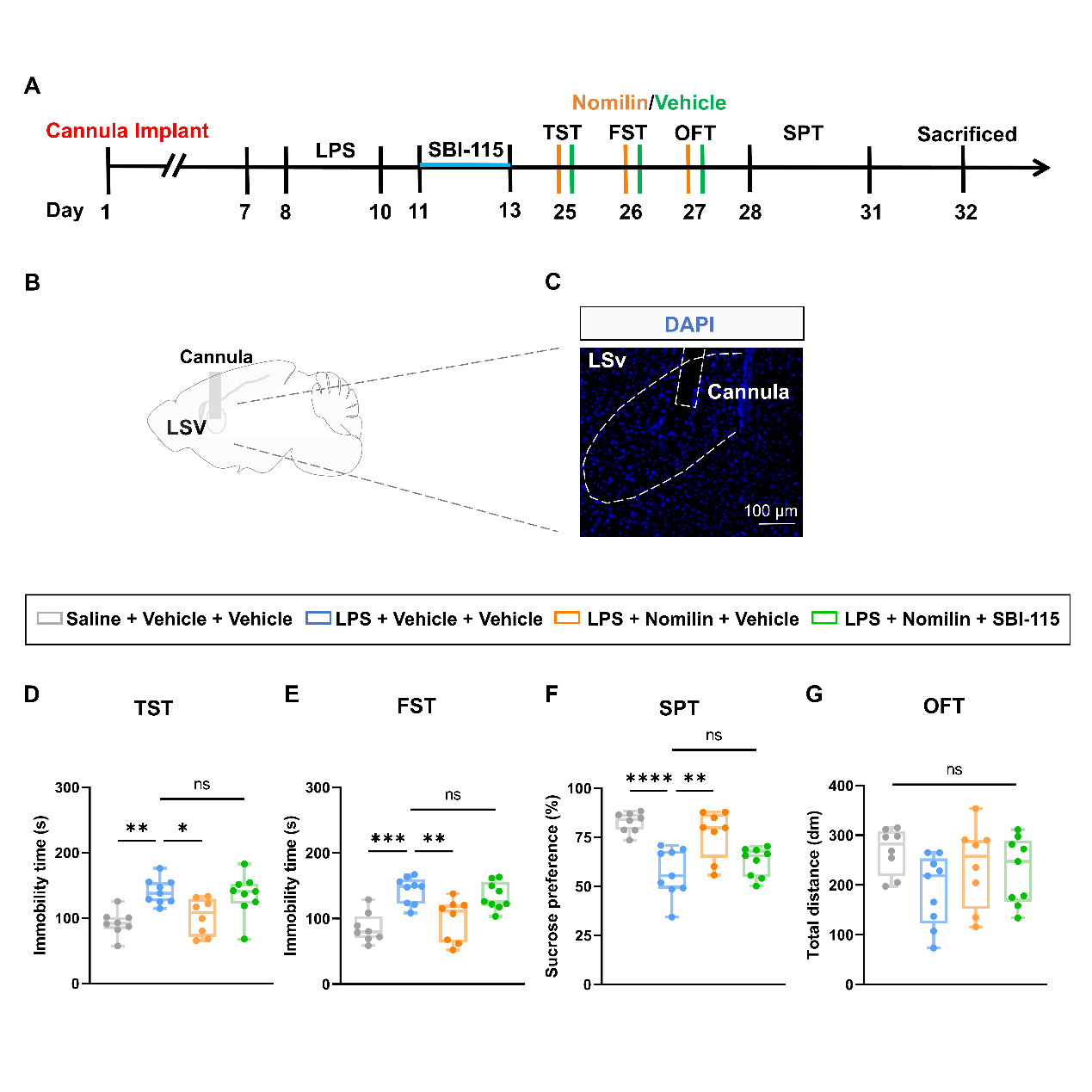


**Figure S4. Nomilin exerts antidepressant effects by stimulating TGR5.**

(A) Experimental design.

(B) Schematic of cannula implantation in the LSv.

(C) Representative image of cannula site in the LSv.

(D) Immobility time in the TST (n = 8–9 mice/group; Two-way ANOVA with Bonferroni's *post hoc* multiple comparisons test; Main effect of nomilin: F_1, 30_ = 0.07969, *P* = 0.7796; Main effect of SBI-115: F_1, 30_ = 22.40, *P* < 0.0001; Interaction: F_1, 30_ = 0.7455, *P* = 0.3947).

(E) Immobility time in the FST (n = 8–9 mice/group; Two-way ANOVA with Bonferroni's *post hoc* multiple comparisons test; Main effct of nomilin: F_1, 30_ = 0.03905, *P* = 0.8447; Main effect of SBI-115: F_1, 30_ = 31.29, *P* < 0.0001; Interaction: F _1, 30_ = 1.597, *P* = 0.2161).

(F) Sucrose preference in the SPT (n = 8–9 mice/group; Two-way ANOVA with Bonferroni's *post hoc* multiple comparisons test; Main effect of nomilin: F_1, 30_ = 0.002691, *P* = 0.9590; Main effect of SBI-115: F_1, 30_ = 35.13, *P* < 0.0001; Interaction: F _1, 30_ = 2.899, *P* = 0.0990).

(G) Total distance traveled in the OFT (n = 8–9 mice/group; Two-way ANOVA with Bonferroni's *post hoc* multiple comparisons test; Main effect of nomilin: F_1, 30_ = 0.03370, *P* = 0.8556; Main effect of SBI-115: F_1, 30_ = 3.616, *P* = 0.0669; Interaction: F_1, 30_ = 2.214, *P* = 0.1472).

Boxplot data are presented as median, quartile, and extremum values. Significance levels: ns *P* > 0.05; ^∗^*P* < 0.05; ^∗∗^*P* < 0.01; ^∗∗∗^*P* < 0.001; ^∗∗∗∗^*P* < 0.0001.


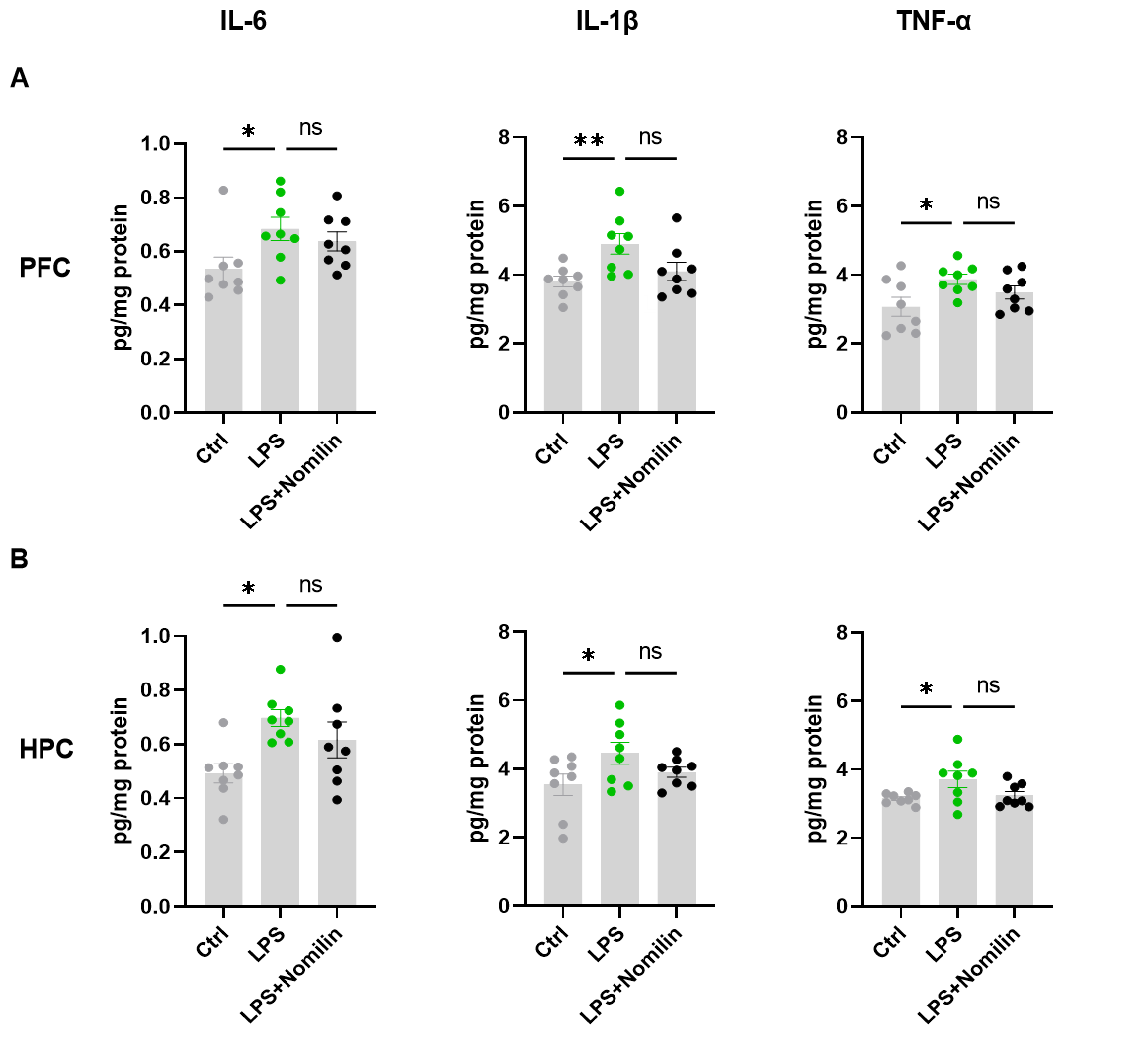


**Figure S5. The antidepressant effects of nomilin have little to do with its anti-inflammatory properties .**

1. The content of inflammatory factors in PFC (n = 8 mice/group; IL-6: Kruskal-Wallis test with Dunn's multiple comparisons test, *P* = 0.0249; IL-1β: One-way ANOVA with Dunnett’s *post-hoc* test, F_2, 21_ = 5.201, *P* = 0.0146; TNF-α: One-way ANOVA with Dunnett’s *post-hoc* test, F_2, 21_ = 3.533, *P* = 0.0476).
2. The content of inflammatory factors in HPC (n = 8 mice/group; IL-6: One-way ANOVA with Dunnett’s *post-hoc* test, F_2, 21_ = 4.793, *P* = 0.0193; IL-1β: One-way ANOVA with Dunnett’s *post-hoc* test, F_2, 21_ = 2.889, *P* = 0.0779; TNF-α: One-way ANOVA with Dunnett’s *post-hoc* test, F_2, 21_ = 3.630, *P* = 0.0442).

Data are presented as mean ± SEM. Significance levels: ns *P* > 0.05; ^∗^*P* < 0.05; ^∗∗^*P* < 0.01.

PFC, prefrontal cortex; HPC, hippocampus.


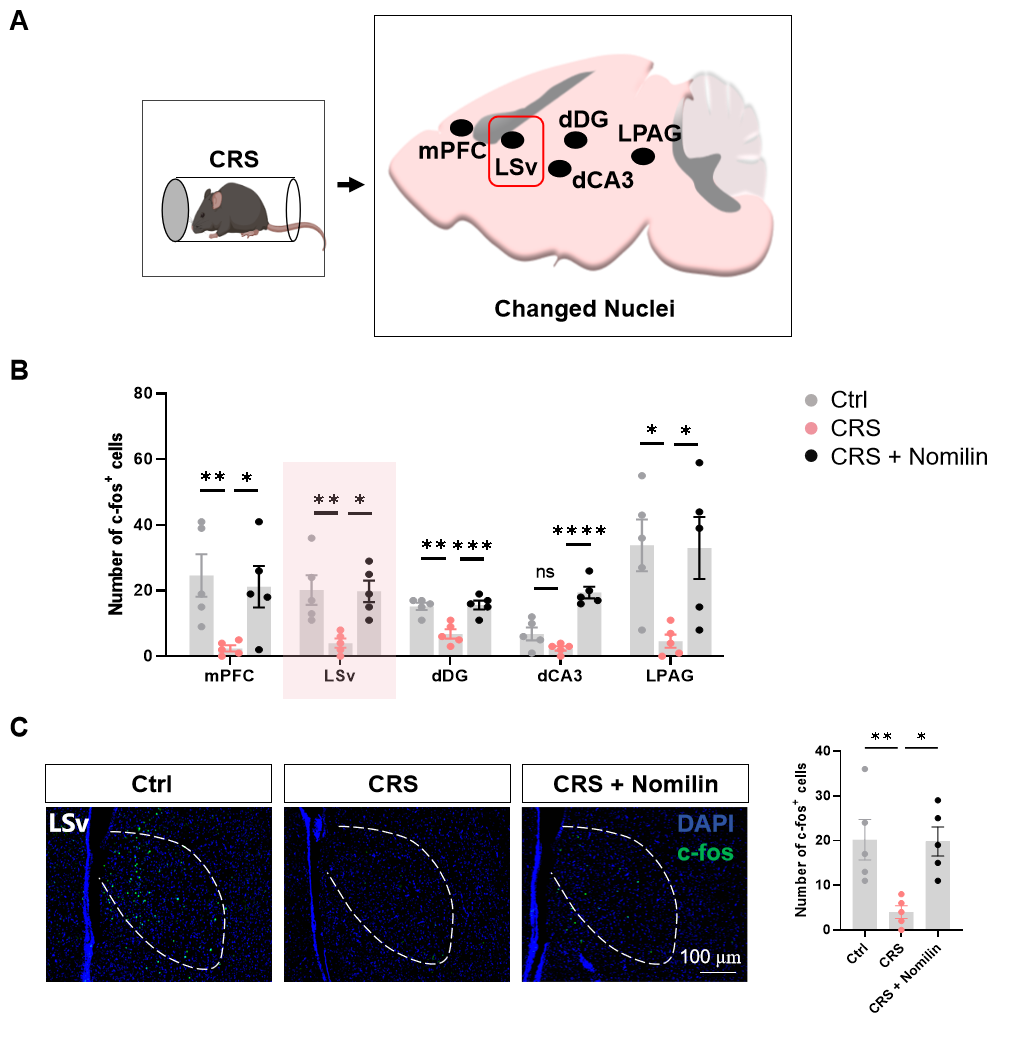
**Figure S6. Nomilin enhances GABAergic neuronal excitability in the LSv of CRS-treated mice.**

(A) Summary plot illustrating changes in c-fos expression across nuclei in CRS-treated mice.

(B) Quantitative analysis of c-fos+ cell counts in different brain regions (n = 5 mice/group; One-way ANOVA with Dunnett’s *post-hoc* test; F_2, 12_ = 5.170, *P* = 0.0240 for mPFC; F_2, 12_ = 7.719, *P* = 0.0070 for LSv; F_2, 12_ = 14.70, *P* = 0.0006 for dDG; F_2, 12_ = 31.73, *P* < 0.0001 for dCA3; F_2, 12_ = 5.330, *P* = 0.0221 for LPAG).

(C) Left: representative images of c-fos expression in the LSv. Right: quantification of c-fos+ cells in the LSv (n = 5 mice/group; One-way ANOVA with Dunnett’s *post-hoc* test, F_2, 12_ = 7.719, *P* = 0.0070).

Data are presented as mean ± SEM. Significance levels: ns *P* > 0.05; *^∗^P* < 0.05; *^∗∗^P* < 0.01; *^∗∗∗^P* < 0.001; *^∗∗∗∗^P* < 0.0001.

dCA3, dorsal hippocampal CA3.


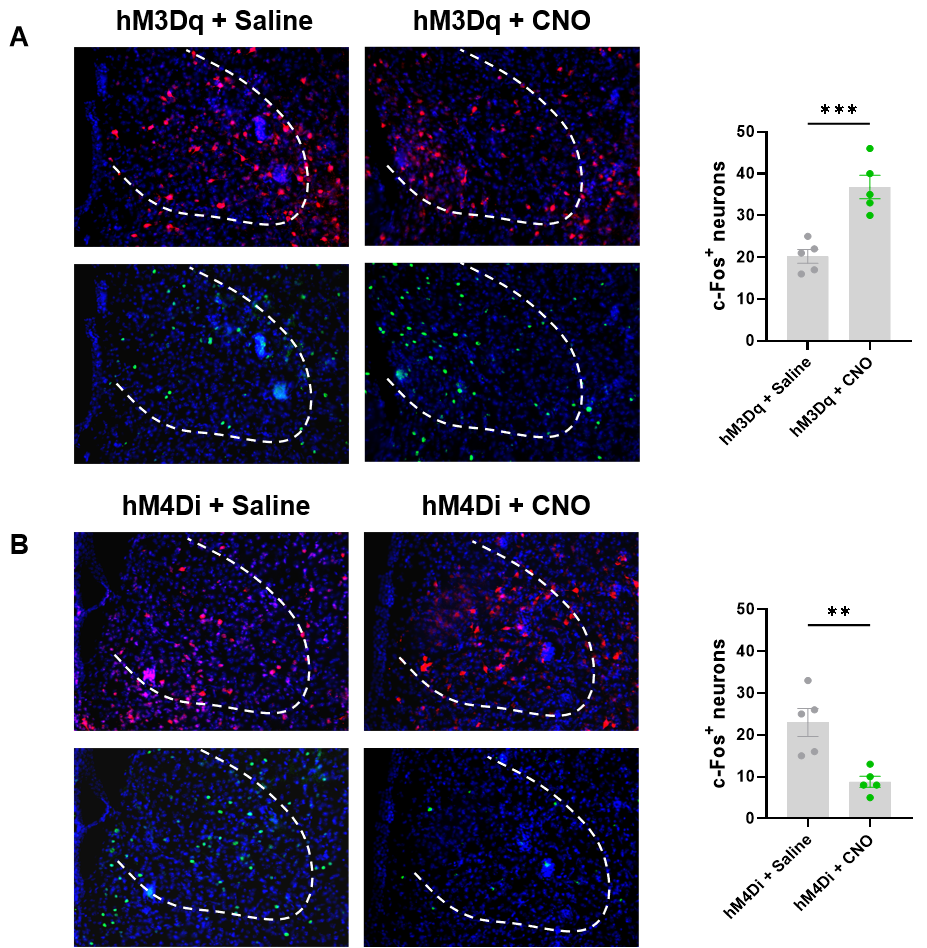
**Figure S7. C-fos staining verifies the validity of chemogenetics.**

1. The expression of AAV-GAD67-hM3Dq-mCherry in the soma of LSv and the activation of LSv GABAergic neurons by hM3Dq significantly increased c-Fos expression induced by CNO (n = 5 mice/group; Two-tailed unpaired Student’s *t*-test; t (8) = 5.080 , *P* = 0.0010).
2. The expression of AAV-GAD67-hM4Di-mCherry in the soma of LSv and the inhibition of LSv GABAergic neurons by hM4Di significantly reduced c-Fos expression induced by CNO (n = 5 mice/group; Two-tailed unpaired Student’s *t*-test; t (8) = 3.932 , *P* = 0.0043).

Data are presented as mean ± SEM. Significance levels: *^∗∗^P* < 0.01; *^∗∗∗^P* < 0.001.


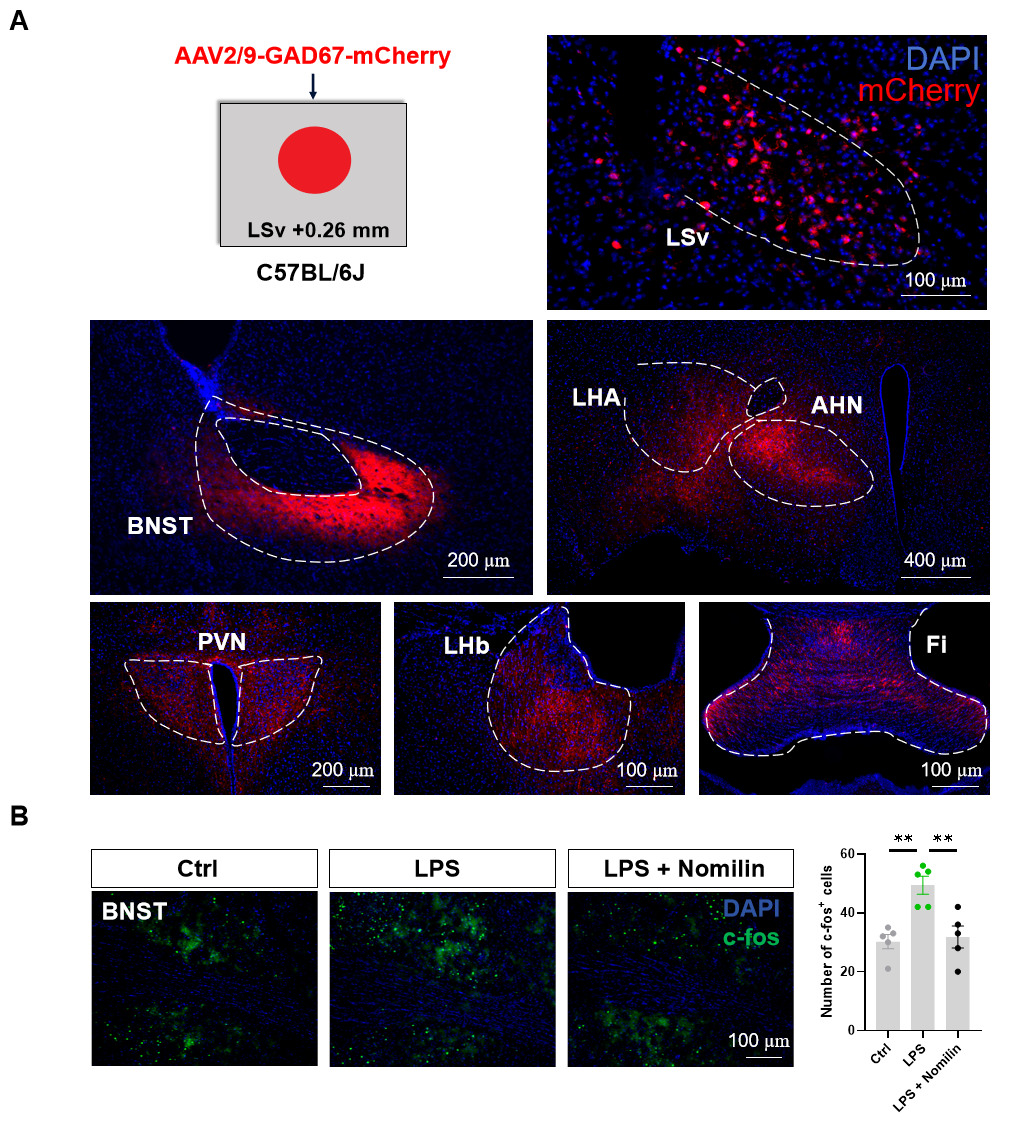


**Figure S8. BNST is a key downstream target of the LSv in the antidepressant effects of nomilin.**

(A) Representative images of axon terminals expressing mCherry in the BNST, LH, AHN, PVN, LHb, and Fi.

(B) Left: representative images of c-fos expression in the BNST. Right: quantitative analysis of c-fos+ cells in the BNST (n = 5 mice/group; One-way ANOVA with Dunnett’s *post-hoc* test, F_2, 12_ = 11.68, *P* = 0.0015).

Data are presented as mean ± SEM. Significance levels: *^∗∗^P* < 0.01.

Fi, fimbria of the hippocampus.
